# The role of atmospheric dynamics and large‐scale topography in driving heatwaves

**DOI:** 10.1002/qj.4306

**Published:** 2022-07-07

**Authors:** Bernat Jiménez‐Esteve, Daniela I.V. Domeisen

**Affiliations:** ^1^ Institute for Atmospheric and Climate Science ETH Zurich Zurich Switzerland; ^2^ Université de Lausanne Lausanne Switzerland

**Keywords:** atmospheric dynamics, blocking, heatwaves, idealized modeling, jet, latitude, topography

## Abstract

Heatwaves are weather events characterized by extreme near‐surface temperature anomalies that persist for several days, and therefore lead to catastrophic impacts on natural ecosystems, agriculture, human health, and economies. Different physical processes can contribute to the temperature anomaly associated with heatwaves. Previous studies have shown that increased solar radiation and adiabatic heating associated with blocking systems and local land–atmosphere coupling are important drivers of summer heatwaves. Less is known about the fundamental role of atmospheric large‐scale dynamics and topography in generating heatwaves. Here, we perform idealized model simulations where all physical parameterisations are substituted by a simple zonally symmetric temperature relaxation scheme. This allows us to characterize the dynamical processes involved in the life cycle of heatwaves occurring at different latitudes. We find that blocking plays an active role in the life cycle of high‐ and midlatitude heatwaves, while blocking is less relevant for low‐latitude heatwaves. Rossby‐wave packets are the dominant drivers for midlatitude heatwaves, with horizontal advection being the main mechanism leading to heat extremes. Heatwaves exhibit a higher persistence and frequency near the pole and Equator compared with the midlatitudes, but a higher intensity in the midlatitudes compared with higher and lower latitudes. Topography located along the midlatitude jet has the largest impact on the heatwave distribution around the planet, resulting in increased heatwave frequency upstream for moderate topographic forcing and a circumglobal increase for topographic elevations above 6 km. Identifying the most relevant processes driving heatwaves can potentially benefit the prediction and representation of extreme events in operational weather and climate forecast systems.

## Graphical abstract

Heatwaves are weather events characterized by extreme near‐surface temperature anomalies that persist for several days. By performing idealized model simulations, we study the underlying role of atmospheric dynamics and topography on the life cycle of heatwaves at different latitudes. We find that the dynamics of midlatitude heatwaves are strongly associated with the zonal propagation of Rossby‐wave packets, while atmospheric blocking dominates at higher latitudes. Topography has an effect on the longitudinal location of heatwaves.

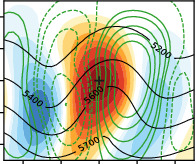

**Abbreviations**
ITCZ: inter‐tropical convergence zone
RWP: Rossby‐wave packet
SST: sea surface temperature

## 1 INTRODUCTION

Heatwaves are near‐surface extreme temperature events that persist for several days, thus causing devastating impacts on human health (including increased mortality), agriculture, and natural ecosystems (Robine *et al*., 2008; García‐Herrera *et al*., 2010; Shaposhnikov *et al*., 2014; Sivakumar, 2020; Kornhuber *et al*., 2020). The physical drivers of heatwaves are diverse and can be classified into two main groups: atmospheric drivers (including moist and radiative processes) and surface drivers. In addition, surface and atmospheric drivers influence each other, which can lead to combined nonlinear effects (for an overview on heatwave drivers, see, e.g., Perkins, 2015; Horton *et al*., 2016; Miralles *et al*., 2019). In the present study, we isolate the contribution from dry atmospheric dynamics to heatwaves using targeted model experiments.

Atmospheric circulation drivers of heatwaves include quasistationary upper‐level ridges linked to surface high‐pressure anticyclones and local amplification of Rossby waves, so‐called Rossby‐wave packets (RWPs: Wirth *et al*., 2018). RWPs have been identified as important drivers for both cold and warm events, as they are also associated with a strong meandering of the upper‐level jet at weather (Fragkoulidis *et al*., 2018) and subseasonal timescales (Wolf *et al*., 2018). High‐pressure anticyclones can develop into atmospheric blocking due to wave breaking and can persist for several days. The effect of blocking on near‐surface temperature varies with the seasonal cycle (e.g., Sousa *et al*., 2018; Nabizadeh *et al*., 2021). During summer, large‐scale subsidence and increased solar radiation associated with clear‐sky conditions within the anticyclone lead to strong near‐surface warming, which in turn increases the likelihood of a heatwave (Pfahl and Wernli, 2012; Schaller *et al*., 2018; Zschenderlein *et al*., 2019; 2020; Li *et al*., 2020; Xu *et al*., 2020). Summer heatwaves are therefore also often associated with weak pressure gradients, that is, a weak large‐scale circulation (Spensberger *et al*., 2020). In winter, enhanced long‐wave radiative cooling during the longer nights and decreased solar radiation within blocking anticyclones lead to near‐surface vertical temperature inversions, separating the cold boundary layer from the free atmosphere, which is heated by large‐scale subsidence (Nabizadeh *et al*., 2021). Thus, in contrast to summer, winter blocking situations are not necessarily associated with extreme warm surface temperatures (e.g., Sousa *et al*., 2018). In this study, we analyse the circulation contribution (excluding radiation and other diabatic effects) in the life cycle and properties of idealized heatwaves at different latitudes.

Heatwaves have also been linked to surface drivers related to energy and momentum exchange at the interface between the atmosphere and the Earth's surface (ocean or land). The role of soil moisture has been studied extensively, and has been shown to modulate the amount of latent and sensible turbulent vertical heat flux, which controls the near‐surface air temperature and can lead to extremely warm and persistent temperature anomalies (e.g., Fischer *et al*., 2007a; 2007b; Hirschi *et al*., 2011; Miralles *et al*., 2012; Rasmijn *et al*., 2018; Wehrli *et al*., 2019). Dry soil conditions limit surface evaporation, leading to a decrease in the latent heat flux, which is compensated by an increased sensible heat flux and thus an increase in air temperature near the surface. In contrast, when the soil is wet, most of the solar radiation reaching the surface is used in evaporation and the sensible heat flux is much weaker, leading to a weaker near‐surface temperature increase (e.g., Miralles *et al*., 2019). Soil moisture can also influence the persistence of heatwaves (Lorenz *et al*., 2010). Land–atmosphere feedbacks are important in summer, when solar radiation is strong, thus the studies cited above focus on summer heatwaves. Here, we isolate the dynamical drivers and therefore the radiative effects and land–atmosphere feedbacks are not simulated in our targeted experiments. Due to the expected importance of local diabatic effects for tropical heatwaves, here we focus on the latitude range 25–65${}^{\circ }N$, separating polar, midlatitude, and subtropical processes.

The distribution and frequency of heatwaves around the globe is modulated further by topography and continental boundaries. Large‐scale topography plays a fundamental role in setting the location of stationary climatological waves (Charney and Eliassen, 1949; Held *et al*., 2002; Chang, 2009; Lutsko and Held, 2016), and thus also shapes the location of atmospheric blocking (Narinesingh *et al*., 2020), storm tracks (Kaspi and Schneider, 2013), and temperature variability (Lutsko *et al*., 2019). It can therefore be expected that large‐scale topography also impacts the frequency and location of heatwaves through changes in the location and strength of the midlatitude jet stream. In this study, we analyse the impact of topography on heatwave distribution and frequency by adding an idealized Gaussian mountain of varying height and latitudinal location in a dry dynamical core simulation. This approach allows us to isolate the role of topography in the absence of complex interactions with the other components of the climate system.

The aim of this study is twofold: first, to understand better the fundamental role of atmospheric dynamics in the evolution of heatwaves, and, second, to assess the basic role of topography and latitude in setting the distribution of these extreme events. We identify and characterize heatwaves occurring in a zonally symmetric dry dynamical core in the absence of physical parameterisations, thus allowing us to isolate the role of atmospheric dynamics in the evolution of heatwaves.

## 2 DATA AND METHODS

### 2.1 Model configuration

We use the ICOsahedral Nonhydrostatic (ICON) atmospheric model, consisting of an unstructured triangular horizontal grid (Zängl *et al*., 2015). We employ a horizontal resolution of $R_{2}B_{4}$, which is equivalent to ∼158 km grid spacing, and 41 vertical levels.

In order to analyse the influence of dry dynamics and the sensitivity to topography on heatwaves, we use an idealized configuration of the model. In this configuration, physical parameterisations are turned off and substituted by a Newtonian temperature relaxation term of the form $Q=-(T-T_{\text{eq}})/\tau$ (Held and Suarez, 1994), where τ is the relaxation timescale and T is the local temperature at a given time step and grid point. $T_{\text{eq}}$ is the reference equilibrium temperature towards which the local temperature is relaxed, as an approximation of what unresolved processes like radiation and moist convection would produce without dynamics. $T_{\text{eq}}(\phi ,p)$ is zonally symmetric, that is, it only depends on latitude ϕ and pressure level p in the following way:

(1) $$\begin{array}{ll} T_{\text{eq}}(\phi ,p) & =\max\left\{200\;K,\left[315\;K-{\left(\Delta T\right)}_{y}\;\sin^{2}\;\phi \right.\right.\\ & \;\left.\left.-{\left(\Delta \theta \right)}_{z}\log\left(\frac{p}{p_{0}}\right)\cos^{2}\;\phi \right]{\left(\frac{p}{p_{0}}\right)}^{\kappa }\right\}, \end{array}$$

where ${\left(\Delta T\right)}_{y}=60$ K and ${\left(\Delta \theta \right)}_{z}=10$ K are the two parameters setting the meridional and vertical equilibrium temperature distribution, respectively. $\kappa =R/C_{p}=2/7$ and $p_{0}=1,000$ hPa are constants. The relaxation timescale τ follows the following distribution:

(2) $$\begin{array}{ll} \tau^{-1}(\phi ,\sigma ) & =k(\phi ,\sigma )\\ & =k_{a}+(k_{s}-k_{a})\max\left(0,\frac{\sigma -\sigma_{b}}{1-\sigma_{b}}\right)\cos^{4}\;\phi , \end{array}$$

where σ=p/(1,000hPa) is the model's terrain‐following vertical coordinate; $k_{a}=1/40\;{\;\text{day}}^{-1}$ and $k_{s}=1/4\;{\text{day}}^{-1}$ are two parameters setting the distribution of the relaxation coefficient. $\sigma_{b}=0.7$ is the vertical model level where the top of the boundary layer is defined. Below this level, a stronger temperature relaxation is applied (Equation (2)). This configuration leads to a climatological state that is close to the observed annual mean or equinox conditions.

**FIGURE 1:**
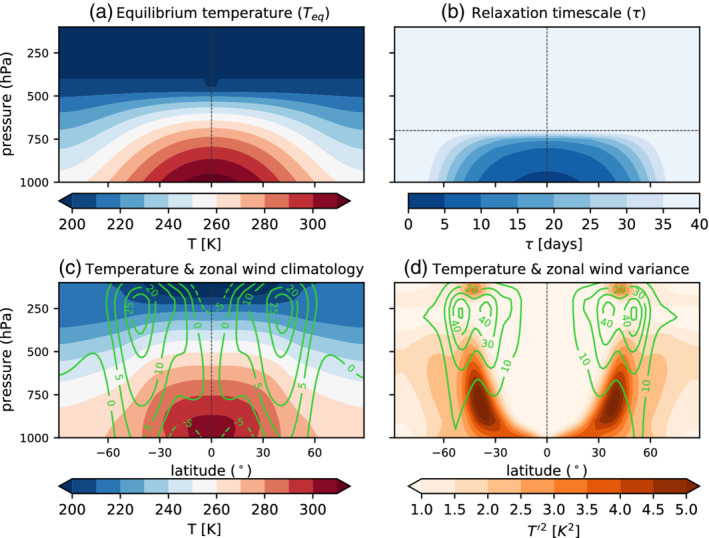
Held–Suarez simulation setup. (a) Equilibrium temperature distribution (calculated from Equation (1)), (b) relaxation time (Equation (2)), (c) zonal mean climatology of temperature (shading) and zonal wind (green contours in units of m·s${}^{-1}$), and (d) monthly temperature (shading) and zonal wind (contours) variance. The horizontal dashed line in (b) represents the parameter $\sigma_{b}=0.7$, which can be interpreted as the top of the boundary layer. The 30‐year period of the no‐topography run is used to compute the model climatology in (c) and (d) [Colour figure can be viewed at wileyonlinelibrary.com]

The distributions of $T_{\text{eq}}$ and τ with latitude and height are shown in Figure 1, together with the model zonal mean climatology of zonal wind U and temperature T. The differences between T and $T_{\text{eq}}$ (compare Figure 1a,c) arise from the poleward transport of heat by both the mean circulation and the eddies, resulting in a weakening of the meridional temperature gradient. Note that this model configuration does not have a representation of the stratosphere. The stratosphere is thought to have a negligible impact on the dynamics of surface heatwaves, although in the Southern Hemisphere some studies point at an impact for extreme temperature events over Australia at subseasonal timescales (Lim *et al*., 2019; Domeisen and Butler, 2020).

To represent boundary‐layer friction, a simple linear Rayleigh damping of horizontal velocities is applied for $\sigma >\sigma_{b}$, as described in Held and Suarez (1994). The damping follows the σ coordinate, so it follows the topography.

### 2.2 Topography sensitivity experiments

The second aim of this study is to analyse the impact of large‐scale topography on the frequency of heatwaves in the idealized model configuration described above. The topographic forcing is added in the model by changing the height of the lowest model level. Specifically, the topography or surface height h is defined analytically as a function of latitude ϕ and longitude λ using the following expression from Cook and Held (1992):

(3) $$h(\phi ,\lambda )=H\exp\left\{-\left[\frac{{\left(\lambda -\lambda_{0}\right)}^{2}}{a^{2}}+\frac{{\left(\phi -\phi_{0}\right)}^{2}}{b^{2}}\right]\right\},$$

where H is the maximum height of a Gaussian mountain and a and b are the zonal and meridional half‐widths of the mountain, respectively. We set

$$a=\frac{1,\;500\;\text{km}}{R_{e}\cos\phi }\;\text{and}\;b=\frac{1,\;500\;\text{km}}{R_{e}},$$

where $R_{e}=6,371$ km is the radius of the Earth, which preserves the size of the mountain when changing its latitude and produces a response that is large enough in magnitude and extent to be resolved in our model (similar to Cook and Held (1992) and Narinesingh *et al*. (2020)). The parameters $\phi_{0}$ and $\lambda_{0}$ set the latitude and the longitude of the mountain peak.

We perform a set of simulations where we vary the latitudinal location ($\phi_{0}=25,45,65^{\circ }$N) and the maximum height (H= 2, 3, 4, 6, 8 km) of the topography systematically, while all the other parameters are kept fixed ($\lambda_{0}=90^{\circ }$E). This leads to a total of 16 model simulations (15 simulations using different topographic configurations and the control run without topographic forcing, referred to as no‐topography). Each simulation is run for a total of 30 years using a constant equilibrium temperature and relaxation timescale (see previous section). The simulations are initialized from an isothermal atmospheric state, and thus the first year is disregarded as spin‐up.

### 2.3 Heatwave identification

There is currently no commonly agreed‐upon definition for heatwaves in the literature. Most definitions include temperature at 2 m exceeding a particular extreme threshold and a persistence criterion, that is, the threshold must be exceeded for several consecutive days (for a review see Perkins, 2015).

Here we identify heatwaves using the 95th percentile of temperature at the 1,000‐hPa pressure level. This threshold is computed independently for each grid point and for the entire model run (note that there is no seasonal cycle). In Section 6.3, the 95th percentile is computed for each day of the year independently for the ERA‐Interim reanalysis (1979–2019: Dee *et al*., 2011). After identifying days exceeding this extreme threshold, a persistence filter of 3 days is applied. We do not consider a minimum distance between heatwave events. Note that even though heatwaves are identified at the interpolated 1,000‐hPa pressure level and not at the first model sigma level, this does not affect our results.

For each heatwave we identify the onset date (first day with temperatures exceeding the 95th percentile), duration (number of days from the onset to the last day exceeding the extreme threshold), mean intensity (averaged daily temperature anomalies during the heatwave period), maximum intensity (the maximum daily mean anomaly during the heatwave event), and mean longitudinal extent (the number of continuous grid points in the zonal direction). The longitudinal extent is transformed to “km” by accounting for the latitude at which the heatwave occurs. Anomalies are computed with respect to the 30‐year climatology of the respective simulation.

To obtain the mean life cycle of a heatwave event, composites of different atmospheric variables (temperature, geopotential height, and wind) are computed for each grid point with respect to the onset date of all heatwaves identified at individual grid points. These life cycles are then averaged for all heatwaves occurring at the same latitude. To account for the fact that many grid points classified as heatwaves are caused by the same synoptic system and are therefore strongly autocorrelated, we compute an equivalent sample size ($N_{e}$). $N_{e}$ is calculated by adding the inverse of all the longitudinal extents (LE), given as a number of grid points, for all the composited heatwave grid points (N):

(4) $$N_{e}=\overset{N}{\underset{i=1}{\sum }}\frac{1}{LE_{i}}.$$

This leads to an estimate of the equivalent sample size of $N_{e}$(ϕ = 65) = 2,697, $N_{e}$(ϕ = 45) = 2,600, $N_{e}$(ϕ = 25) = 4,442. These sample sizes are around 5–10 times smaller than the number of heatwave grid points used for the composite, but still sufficiently large to guarantee the statistical robustness of the results.

### 2.4 Atmospheric blocking index and Rossby‐wave envelope calculation

Atmospheric blocking is detected on a grid‐point basis. First, we interpolate the model data to a regular horizontal grid of $2.5^{\circ }\times 2.5^{\circ }$ to be consistent with previous blocking studies. We follow the blocking‐index definition used in Davini *et al*. (2012), which is based on the meridional reversal of the geopotential height gradient at 500 hPa, following the original one‐dimensional index by Tibaldi and Molteni (1990) and Scherrer *et al*. (2006).

We perform this analysis for the Northern Hemisphere. For every longitude/latitude grid point located between 30 and 75°N $\left(\lambda_{0},\phi_{0}\right)$, we define the geopotential height gradient towards the south (GHGS) and geopotential height gradient towards the north (GHGN) as

(5) $$\begin{array}{ll} GHGS(\lambda_{0},\phi_{0}) & =\frac{Z500(\lambda_{0},\phi_{0})-Z500(\lambda_{0},\phi_{S})}{\phi_{0}-\phi_{S}}, \end{array}$$

(6) $$\begin{array}{ll} GHGN(\lambda_{0},\phi_{0}) & =\frac{Z500(\lambda_{0},\phi_{N})-Z500(\lambda_{0},\phi_{0})}{\phi_{N}-\phi_{0}}, \end{array}$$

where $\phi_{0}$ ranges from 75–30°N, $\phi_{S}=\phi_{0}-15^{\circ }$, and $\phi_{N}=\phi_{0}+15^{\circ }$.

An instantaneous blocked grid point is identified when the daily mean geopotential height gradient reverses ($GHGS(\lambda_{0},\phi_{0})>0$) and the northern gradient is weak enough ($GHGN(\lambda_{0},\phi_{0})<-10\;m$ per degree latitude). Additionally, the Z500 anomaly for a blocked grid point has to be above an amplitude threshold, computed as the 90‐day running mean of the daily 90th percentile of Z500 anomalies over 20–90°N. Note that this criterion applies only to the instantaneous blocked grid point, rather than the full latitude range used to compute the threshold value. The last condition ensures that the blocking anomaly is also related to large‐scale geopotential height anomalies (note that this is different from the definition used in Davini *et al*. (2012), but is similar to Dunn‐Sigouin and Son (2013)). Imposing the amplitude threshold reduces the amount of subtropical blocking that is associated with weak Z500 anomalies.

Additionally, a minimum longitudinal extent of $15^{\circ }$ is required to ensure the extent usually associated with a blocking event (Davini *et al*., 2012). Note that imposing a minimum longitudinal extent also guarantees the latitudinal extent of the blocking event. Finally, to ensure persistence, a grid point is defined as being blocked only when blocking occurs within two longitude (5°) or one latitude (2.5°) grid points around each grid point for at least five consecutive days. Varying the longitudinal extent ($\pm 2.5^{\circ }$) or persistence (±1 day) requirement slightly does not yield qualitatively different results. The annual mean climatology of blocking frequency using this definition is displayed in the Supporting Information in Figure S1 for ERA‐Interim (1979–2019), the control run (no‐topography), and the run using a 4‐km mountain centered at 45°N.

In order to study the dynamical connection between heatwaves and Rossby waves, the Rossby‐wave packet envelope, that is, a measure of the local wave amplitude, is calculated following Fragkoulidis *et al*. (2018). Here we use the daily mean anomalies (instead of six‐hourly instantaneous values as in Fragkoulidis *et al*., 2018) of the meridional wind component at 300 hPa (V300). The anomalies are computed with respect to the model climatology, which has no seasonal cycle. We retain only synoptic‐scale zonal wavenumbers (k=4–8) by using a Fourier transformation in the longitudinal direction prior to the calculation of the wave envelope. The RWP envelope is calculated separately for each latitude band and each time step using a Hilbert transform method following Zimin *et al*. (2003). Expanding the zonal wavenumber range (k=4–10) does not lead to qualitatively different results, as most of the wave spectrum is concentrated around synoptic wavenumbers (k=4–8).

### 2.5 Local temperature tendency analysis

To quantify the physical processes that lead to local changes of temperature from an Eulerian perspective, we employ the temperature tendency equation in the following form:

(7) $$\frac{\partial T}{\partial t}=-u\frac{\partial T}{\partial x}-v\frac{\partial T}{\partial y}-\omega \frac{\partial T}{\partial p}+\frac{1}{\rho C_{p}}\omega +Q,$$

where the left‐hand side term represents the local temporal rate of change of temperature T(x,y,z,t). The first two terms on the right‐hand side represent horizontal temperature advection, the third term is vertical advection, the fourth term is adiabatic warming/cooling, and Q represents the diabatic heating of the air, which represents radiative processes as well as turbulent latent and sensible heat fluxes.

We compute each term of the equation using daily mean values and centered finite differences on an interpolated pressure level and regular grid with a $2.5\times 2.5^{\circ }$ horizontal resolution. The term Q is estimated as a residual in Equation (7), assuming local temperature balance. A detailed description of each term in this equation, together with the model climatology, can be found in the supplementary material.

To analyse the contribution of the anomaly and climatological terms further, we linearize Equation (7) with respect to the background climatology following Tamarin‐Brodsky *et al*. (2019). This is done by decomposing the temperature and the three wind components into their climatology and anomaly parts: $T=T_{c}+T_{a}$, (u,v,ω) = ($u_{c}+u_{a},v_{c}+v_{a},\omega_{c}+\omega_{a}$). More details about the methodology can be found in the supplementary material. The results using this decomposition are shown in the Supporting Information in Figures S6, S7, and S8.

In the Held–Suarez experiments, $Q=-(T-T_{\text{eq}})/\tau$, and thus this term always acts to dampen heat extremes ($T\gg T_{\text{eq}}\to Q<0$), which is different from observed summer heatwaves, where this term can represent strong heating from the ground during summer days with strong insolation. Nevertheless, our model simulations can be used to assess the relative role of subsidence and horizontal advection in generating warm extremes.The lack of realism in the diabatic heating is also the main reason for excluding heatwaves occurring in the Tropics and for focusing on heatwaves occurring between the subtropics and the polar areas [25–$65^{\circ }$], where adiabatic and advective processes play a major role (Figures S2 and S3).

## 3 ZONAL MEAN CLIMATOLOGY OF THE IDEALIZED DRY SIMULATION

**FIGURE 2:**
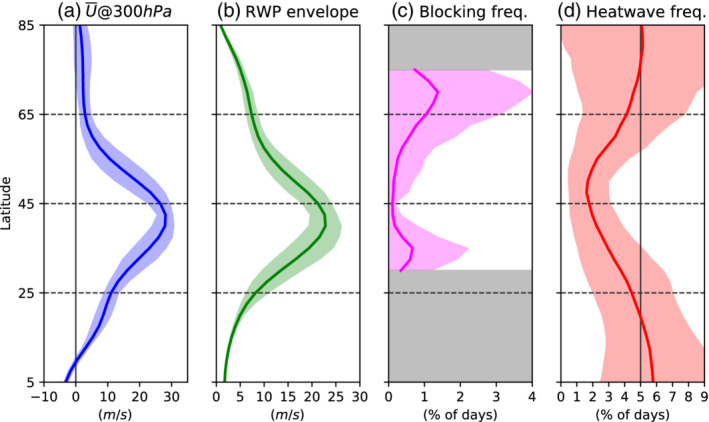
Zonal mean climatology of (a) zonal wind at 300 hPa (U300), (b) Rossby‐wave packet (RWP) envelope at 300 hPa (calculated from the meridional wind following Fragkoulidis *et al.*, 2018), (c) blocking frequency, and (d) heatwave frequency. The semitransparent shading shows the 10th–90th interpercentile range of monthly variability. Note that the blocking frequency is only defined for the latitude band 30–75°N, indicated by the area between the bands of gray shading [Colour figure can be viewed at wileyonlinelibrary.com]

Before analysing the life cycle of heatwaves, we discuss the climatological circulation characteristics of the idealized control run (Figure 2). For reference, the same analysis is reproduced for reanalysis in Figure S4.

The control run is characterized by a single tropospheric jet centered in the midlatitudes (∼45°N) with a peak intensity of ∼30 m·s${}^{-1}$ (Figure 2a), slightly stronger than the zonal average of the observed Northern Hemisphere annual mean climatology (Figure S4). The mean RWP amplitude zonal mean climatology follows a similar latitude dependence, with the strongest wave amplitudes located along the jet axis (Figure 2b). This is consistent with the nature of RWPs, which originate in areas where the potential vorticity (PV) gradient is strongest, that is, along the jet axis (Wirth *et al*., 2018).

Blocking occurs mostly north of the climatological tropospheric jet (Figure 2c), that is, subpolar blocking. A secondary blocking maximum is located around 30°N, which corresponds to low‐latitude or subtropical blocking, which is less able to block the mean flow and tends to be associated with weaker geopotential height anomalies (not shown). It is important to note that the blocking frequency in the model (Figure 2c) is lower than that in the reanalysis (Figure S4), due to the lack of topographic forcing (Narinesingh *et al*., 2020) or the lack of diabatic processes (Pfahl *et al*., 2015; Steinfeld *et al*., 2020), which are not represented in our idealized atmospheric run. Nevertheless, our model simulates the main dynamical features of the midlatitude large‐scale circulation, for example, baroclinic eddies and blocking, which allows us to study their role in generating persistent warm extremes, that is, heatwaves.

The daily frequency of heatwaves (Figure 2d) exhibits a local minimum at the poleward flank of the midlatitude jet (approx. 2% of the days are heatwaves), while the frequency increases towards the pole and Equator, where more than 5% of the days are heatwave days. This latitude dependence of the heatwave frequency is also observed in reanalysis, especially in the Southern Hemisphere (see Figure S4). The intensity and location of the jet stream are thought to be important features that modulate both the frequency and the properties of heatwaves (e.g., Mahlstein *et al*., 2012), which also exhibit important month‐to‐month variability (10th–90th interpercentile range shown as shading in Figure 2d). At the end of the next section, we analyse in more detail the statistical characteristics of heatwaves at different latitudes (duration, intensity, and extent).

## 4 THE ROLE OF LATITUDE IN THE LIFECYCLE AND CHARACTERISTICS OF HEATWAVES

The idealized dry dynamical model simulation with no topography allows us to characterize the typical evolution of heatwaves at different latitudes, independent of any surface forcing or zonal asymmetry. In particular, we are interested in answering the following questions: are heatwaves driven entirely by atmospheric dynamics different in the polar, midlatitude, and subtropical regions, and what are the main differences in their dynamical evolution?

**FIGURE 3:**
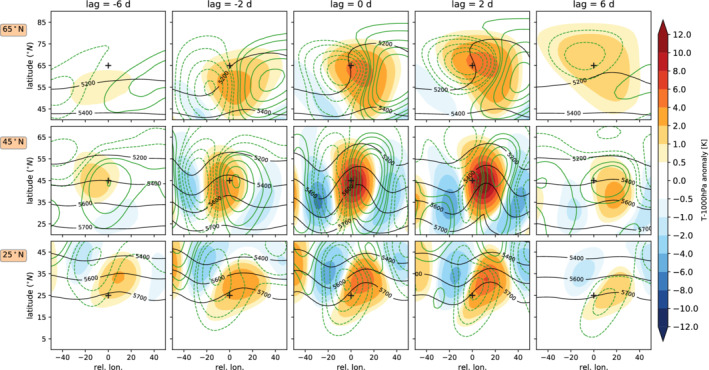
Averaged life cycle of heatwaves in the Held–Suarez configuration, for heatwaves occurring at (top row) 65°N, (middle) 45°N, and (bottom) 25°N. Heatwaves are detected at all grid points at a specific latitude based on the definition found in the methods section. We composite temperature anomalies at 1,000 hPa (color shading), geopotential height anomalies at 1,000 hPa (green contours), and total geopotential height at 500 hPa (black contours) with respect to grid points experiencing a heatwave at different time lags. Crosses in the middle of each panel indicate the coordinates where the heatwave onset is detected at lag 0 [Colour figure can be viewed at wileyonlinelibrary.com]

Figure 3 displays the composite temporal evolution of a heatwave event occurring at three different latitudes in the Held–Suarez relaxation experiment with no topography (see Section 2.1 for details). We composite surface temperature and geopotential height anomalies with respect to the onset of all the heatwaves occurring at all longitudinal grid points at the following latitudes: 65°N, subpolar; 45°N, midlatitudes; 25°N, subtropics. The lags are representative of the developing phase (lag = −6 days), just before onset phase (lag = −2 days), onset (lag = 0), start of decay phase (lag = 2), and post‐heatwave phase (lag = 6). Note that, due to the three‐day minimum persistence criterion, all heatwaves start at lag 0 and persist at least until lag 2. Note that an aspect ratio of 1:2 is used in Figure 3 to visualize the wave‐like patterns better in the longitudinal direction.

**FIGURE 4:**
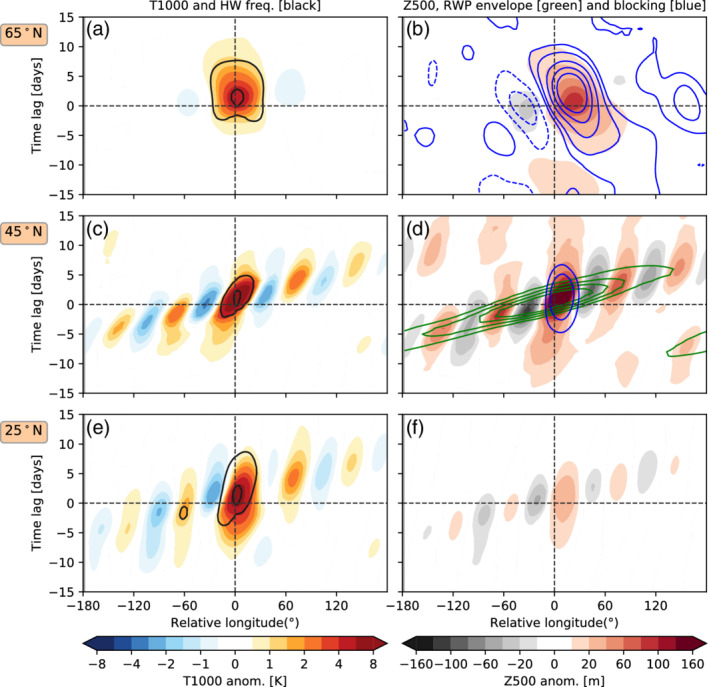
Hovmöller diagram (longitude–time evolution) of temperature anomalies at 1,000 hPa (color shading) and heatwave occurrence (contours: 10, 50% of days) averaged over $\pm 5^{\circ }$ latitude and with respect to the onset date of all heatwaves occurring at (a) 65°N, (c) 45°N and (e) 25°N. (b,d,f) Same as (a,c,e) but for geopotential height anomalies at 500 hPa (Z500), blocking frequency anomalies in % of days (blue contours: −0.5,−0.25 (dashed), 0.25, 0.5, 1, 1.5, 2 (solid)), and RWP envelope (green contours: 22, 24, 26, 28 m·s${}^{-1}$). Note that, when contours are not displayed, this is because the values lie below the smallest displayed contour level. Dashed black lines display the onset and the longitude location where heatwaves are identified at their onset [Colour figure can be viewed at wileyonlinelibrary.com]

We first focus on the average temporal evolution or life cycle of midlatitude heatwaves (center row in Figure 3). The life cycle is characterized by the passage of an eastward‐propagating Rossby‐wave packet that amplifies locally. This is in agreement with the results of Fragkoulidis *et al*. (2018), who find a significant link between extreme temperature events and upper‐level RWPs. RWPs are series of low‐ and high‐pressure centers that do not extend to the full latitude circle at a given time. A wave train extending around the full latitude circle is known as a circumglobal wave pattern (Branstator, 2002; Teng and Branstator, 2019). Previous studies by Petoukhov *et al*. (2013) and Kornhuber *et al*. (2019; 2020) have associated such circumglobal wave patterns with extreme weather and heatwave occurrence. However, when we extend the range of the relative longitude in Figure 3 all the way from −180° to 180° (cf. Figure 4c,d), we do not observe a mean signal of a quasistationary circumglobal pattern. Nevertheless, such resonance events are rare in the real world and are therefore not likely to dominate our composite model analysis either. The effect of higher blocking probability can be observed as positive surface geopotential height anomalies collocated over the heatwave region 6 days before the onset of the heatwave event (Figure 3, middle row). During the onset of idealized midlatitude heatwaves, surface winds tend to be anomalously poleward and extend into the Tropics (20° south), advecting warm air from tropical latitudes (seen as the anomalous geopotential height gradient at 1,000 hPa in Figure 3, middle panel). This anomalous poleward surface wind suggests that heatwaves in this idealized configuration are driven mainly by horizontal warm air advection. Warm air anomalies propagate eastward with time, following the propagation of the upper‐level Rossby wave. Temperatures peak around the onset date, with temperature anomalies surpassing 10 K. Surface temperature anomalies decay quickly after 6 days and propagate equatorward and eastward.

**FIGURE 5:**
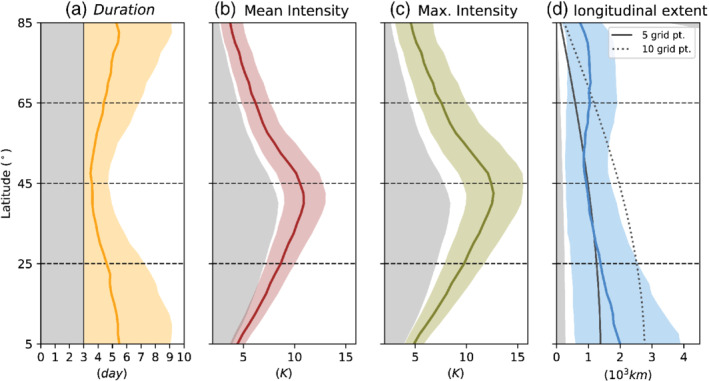
Latitude dependence of heatwave properties in the Held–Suarez no‐topography model experiment: (a) duration (days), (b) mean intensity (K), (c) maximum intensity (K), and (d) longitudinal extent ($10^{3}$ km). The solid line denotes the averaged value and the color shading the 10th–90th interpercentile range for all heatwaves identified at each location. The gray shading in (a) corresponds to the minimum duration of 3 days required for all events, that in (b,c) to the daily 95th percentile of the temperature anomaly for each latitude circle, and that in (d) to the minimum extent (one grid point of 2.5°). To account for the spherical geometric effect, the continuous and dotted gray lines in (d) represent the extent of 5 and 10 grid‐point heatwaves, respectively [Colour figure can be viewed at wileyonlinelibrary.com]

Subpolar or high‐latitude heatwaves (top row in Figure 3) are also associated with poleward surface winds. There are, however, some noticeable differences in their evolution with respect to midlatitude heatwaves. In our idealized model simulations, these temperature extremes tend to be associated with cyclonic wave breaking (see black line in Figure 3). Cyclonic wave breaking is associated with blocking upstream and north of the heatwave region. This is consistent with the results of Masato *et al*. (2013), who find that high‐latitude blocking over Greenland and the North Pacific tends to be associated with cyclonic wave breaking, which leads to significant surface warming. High‐pressure anomalies associated with blocking tend to persist east of the heatwave region from the developing phase of the heatwave and the start of the decaying phase (lag >2 days). During the decaying phase, surface low‐pressure anomalies tend to persist longer than 6 days after the onset of the heatwave. The observed life cycle is consistent with the fact that high‐latitude heatwaves tend to last longer and are less intense than midlatitude heatwaves in our model simulations (cf. Figure 5a,b).

Finally, the mean temporal evolution of subtropical heatwaves is comparable to that of midlatitude heatwaves (Figure 3, bottom row). One important difference is that, due to the more equatorward location, temperature anomalies tend to reach lower values than midlatitude heatwaves (cf. Figure 3, middle versus bottom row), as the advection is proportional to the temperature gradient. At 25°N, heatwave evolution is also associated with the eastward propagation of a RWP, but the maximum of the RWP is located further poleward than the heatwave and along the upper‐level jet. This type of heatwave is related to the deepening and equatorward propagation of an anomalous surface low‐pressure center (dashed green lines in Figure 3, bottom row), which is in a favorable position to advect warmer air from the equatorial region. Surface low pressure tends to persist during the decay phase of the event. This is in contrast with heatwaves occurring 20° further north, which are associated with collocated high‐pressure anomalies in the developing and decaying phases, typically associated with enhanced blocking probability.

Another way to look at the mean life cycle of idealized heatwaves occurring at different latitudes is by using a Hovmöller diagram (Figure 4). In this figure, we have extended the relative longitude coordinate with respect to the center of the heatwave to the full longitude circle [180°W, 180°E]. This allows us to investigate when and where the circulation anomalies that lead to heatwave events originate within the same latitude.

For high‐latitude heatwaves (Figure 4a,b), the temperature anomalies at the 1,000‐hPa isobaric level only emerge five days before the heatwave onset, consistent with the 2D evolution in Figure 3. The 500‐hPa level evolution is characterized by the development of a high‐pressure system east of the heatwave location and is associated with a significantly enhanced blocking probability. This enhanced blocking probability starts developing approximately 10 days before the onset of the event, clearly before the surface temperature anomalies, and on average persists more than 10 days after the onset of the heatwave, while tending to propagate slowly westward until dissipation (Figure 4b).

In contrast, the Rossby‐wave train associated with the evolution of midlatitude heatwaves can develop more than 10 days before the onset of the event (Figure 4c,d). Rossby‐wave trains are identified as high values of the RWP envelope, shown in green contours in Figure 4d. Note that RWP envelope contours are not displayed for small values, below 22 m·s${}^{-1}$, but Z500 anomalies suggest where weaker RWP could be. The RWP associated with heatwave occurrence can develop more than 270° upstream of the heatwave location and propagate more than 360° around the same longitude circle. Thus this diagram confirms that heatwaves occurring in the Held–Suarez no‐topography simulation are mainly linked to eddies propagating along the jet stream and not to stationary‐wave anomalies. Furthermore, locally enhanced blocking probability is observed prior to the arrival of the RWP (Figure 4d), with quasistationary positive Z500 anomalies arising more than 10 days before the heatwave onset.

The temporal evolution of subtropical heatwaves is characterized by a lack of blocking anomalies (note blocking is only defined poleward of 30°), although a weak upper‐level ridge east of the region is a common feature (Figure 4f). Figure 4e also shows a higher stationary behavior of temperature anomalies (they appear a bit less tilted in the Hovmöller diagram than for midlatitude heatwaves) and thus heatwaves here tend to last longer on average (cf. Figure 5a). The RWP slower zonal phase speeds and group velocities might be linked to weaker upper‐level zonal winds at this latitude (e.g., Fragkoulidis and Wirth, 2020).

We have shown that the latitude where heatwave events occur in the idealized model determines their dynamical evolution. Now we characterize the heatwaves according to their duration, mean intensity, maximum intensity, and longitudinal extent, and how these properties depend on the latitudinal location (Figure 5). Consistent with their observed dynamical evolution (Figures 3 and 4), heatwaves in the midlatitudes tend to have a shorter duration than polar and subtropical heatwaves (Figure 5a). On average, heatwaves at $45^{\circ }$N last less than four days, but heatwaves occurring north and south of $65^{\circ }$N and $25^{\circ }$N last on average more than five days. Almost an opposite dependence with latitude is observed for the mean and maximum intensity of heatwave events (Figure 5b,c). Due to the higher variability associated with the jet stream, the 95th percentile threshold used to identify heatwaves (shown as gray shading) is higher at $45^{\circ }$N than in the subtropical and subpolar regions. On average, heatwaves at $45^{\circ }$N exhibit a mean intensity above 10 K, while heatwaves occurring north and south of $65^{\circ }$N and $45^{\circ }$N, respectively, exhibit mean intensities around 5–8 K (Figure 5b). Finally, heatwaves tend to have a mean extent of around 1,000 km, which is approximately five grid points ($2.5^{\circ }\times 2.5^{\circ }$) in the midlatitudes ($45^{\circ }$N), while on average they have a larger extent near the Tropics (Figure 5d). This behavior is consistent with the Rossby deformation radius being larger near the Tropics.

**FIGURE 6:**
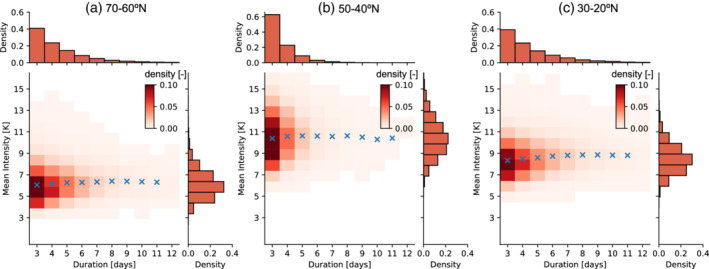
The two‐dimensional probability density function for the duration and intensity of all heatwaves occurring in the regions (a) 70–60°N, (b) 50–40°N, and (c) 30–20°N in the no‐topography simulation. The respective one‐dimensional histograms of duration and intensity are shown on the top and right axes. The blue crosses in each panel indicate the averaged value of the mean intensity for all heatwaves with the same duration [Colour figure can be viewed at wileyonlinelibrary.com]

We also analyse the relationship between the intensity and duration of these idealized dynamical heatwaves. Figure 6 displays the two‐dimensional histogram of the duration and intensity of heatwaves, in combination with the respective one‐dimensional histograms. This representation shows that, while most heatwaves tend to last just three days (60–40% depending on the latitude), there is no apparent statistical relationship between duration and the averaged mean intensity for the heatwaves identified (see blue crosses in Figure 6). This is an interesting result, as, in observed summer heatwaves, larger intensities tend to be associated with more persistent events. Whether the previous statement is true for more complex model configurations would have to be confirmed. Also interesting is the fact that heatwaves in the midlatitudes exhibit a larger variability in their intensity compared with subtropical or subpolar heatwaves, but subtropical and subpolar heatwaves exhibit larger variability in their duration (Figure 6a,c). Note that, when using maximum intensity instead of mean intensity, a very weak link is found with heatwave duration in our idealized simulation (Figure S5). A possible explanation is that for longer‐lasting heatwaves there is a higher probability that a single day during the heatwave exhibits larger temperature anomalies, due simply to natural variability and autocorrelation. However, this effect is rather small: we see a mean increase of 1–2 K in maximum intensity between shorter (3 days) and longer heatwaves (11 days).

## 5 CONTRIBUTIONS TO TEMPERATURE TENDENCIES DURING HEATWAVES

**FIGURE 7:**
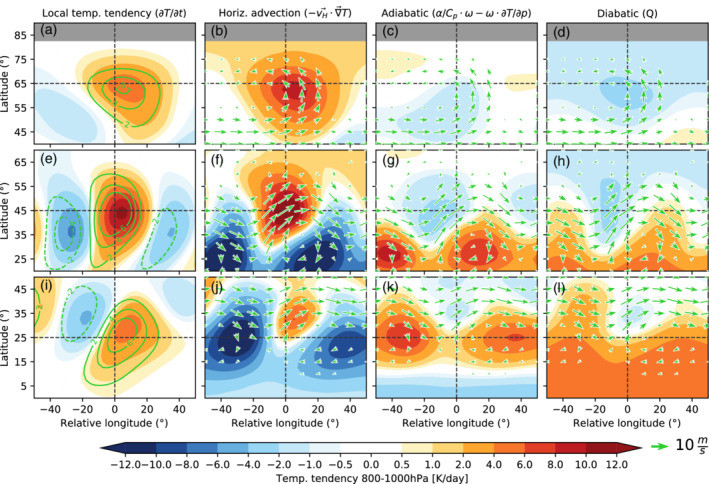
Heatwave‐centered composites of the temperature tendency equation terms (Equation (7)) for all heatwaves identified occurring at (a–d) 65°N, (e–h) 45°N, and (i–l) 25°N. Panel titles indicate the name of the term. Each term is averaged from 800–1,000 hPa and integrated from 6 days before the onset of the heatwave until the onset (lag = 0 days). Green contours in (a,e,i) represent the temperature anomaly in K for the same layer at the onset of the heatwave (contour interval 2 K, negative contours are dashed; the zero contour is left out for clarity). Green arrows in the other three columns represent the wind direction and magnitude at 850 hPa at lag = 0 [Colour figure can be viewed at wileyonlinelibrary.com]

In order to quantify which atmospheric processes lead to the extreme temperature increase associated with heatwaves, we composite the different terms of Equation (7) with respect to the onset and location at which heatwaves occur. Figure 7 displays the three main terms of the temperature tendency equation (Equation (7)), integrated for the six days before onset (from lag −6 to 0 days) and averaged between 800 and 1,000 hPa for all heatwaves occurring at different latitudes in the no‐topography experiment. The temperature anomaly at the onset of these events (green contours in Figure 7a,e,i) and the integrated temperature tendency for this six‐day period (shading in the same panels) show a similar pattern and magnitude, thus most of the temperature increase occurs within this six‐day period. Note that this behavior does not exclude the possibility of remote processes leading to the genesis of heatwave events, which can originate much earlier at another location and propagate to the location where the heatwave is identified: for example, the wave trains shown in Figure 4. A decomposition of the advection and adiabatic terms into climatological, linear, and nonlinear terms is also presented in the supplementary material (Figures S6–S8).

Horizontal temperature advection is the dominant mechanism contributing to the occurrence of heatwaves (Figure 7b,f,j), especially for mid‐ and high‐latitude heatwaves. For midlatitude heatwaves, lower tropospheric (850‐hPa) winds from the southwest bring warmer air from subtropical regions (Figure 7f). For high‐latitude heatwaves the wind comes more directly from the south and turns towards the west at the northern edge of the heatwave center (Figure 7b). This wind pattern is consistent with the cyclonic Rossby‐wave breaking pattern observed in Figure 3 (see figure 1 in Song *et al*., 2011 for comparison). A more in‐depth analysis indicates that nonlinear horizontal advection dominates for heatwaves occurring in the high latitudes, while linear advection dominates for midlatitude and subtropical heatwaves (Figure S8a,b). Adiabatic warming due to large‐scale subsidence is stronger south of the heatwave center for midlatitude heatwaves (Figure 7g). For subtropical heatwaves, adiabatic warming dominates upstream and downstream of the heatwave location (Figure 7k). Thus, although subsidence does not occur co‐located with the heatwave, adiabatically warmed air is advected horizontally to the heatwave location, as indicated by the direction of the 850‐hPa wind.

The diabatic term (represented as a relaxation term in our model simulations) acts as a damping term, as it is proportional to the difference between the actual temperature and the constant equilibrium temperature (Equation (1)). Thus surface interaction or radiative feedback effects that can play an important role in observed heatwaves are not represented in our idealized model simulations. Our model simulations isolate the pure contribution from dry dynamics to persistent heat extremes. Thus, in our composites, diabatic heating is strongest near the Equator and negative near the pole, maintaining the Equator‐to‐pole temperature difference.

**FIGURE 8:**
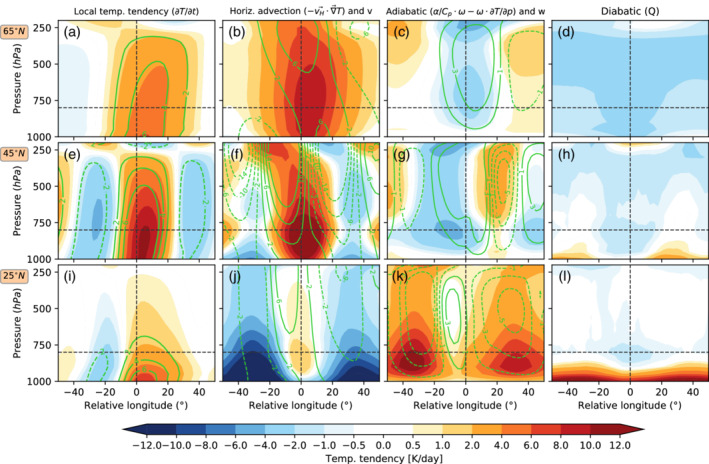
Similar to Figure 7, but showing the longitude–pressure cross‐section centered on the heatwave grid point. Green contours in (b,f,j) display the meridional wind (v) in m·s${}^{-1}$, and in (c,g,k) the vertical velocity (w) in mm·s${}^{-1}$ at the onset of the heatwave [Colour figure can be viewed at wileyonlinelibrary.com]

To understand the vertical structure of the processes leading to heatwaves, Figure 8 displays the cross‐sections of the temperature tendency terms as in Figure 7. The temperature increase is generally strongest close to the surface for all heatwaves (Figure 8a,e,i). However, while the temperature tendency extends to the entire troposphere for high‐ and midlatitude heatwaves (Figure 8a,e), they are more strongly concentrated near the surface for subtropical heatwaves (Figure 8i). Positive horizontal advection is strongest in the lower troposphere for high‐ and midlatitude heatwaves, with a peak around 850 hPa (Figure 8b,f), while the negative advection associated with northerly winds in the subtropics peaks near the surface (Figure 8j). For subtropical heatwaves, adiabatic warming peaks at 900–800 hPa (Figure 8k), and higher for mid‐ and high‐latitude heatwaves (Figure 8c,g). In general, adiabatic warming is also strongest east of high‐pressure systems where subsidence is located (negative w in Figure 8c,g,k) and in the subtropics, where climatological subsidence is strongest (Figures S2b and S7j). This result is consistent with the adiabatic heating associated with extratropical blocking found by Nabizadeh *et al*. (2021). In contrast, positive diabatic warming is confined close to the surface for subtropical heatwaves (Figure 8l) in the idealized model run.

**FIGURE 9:**
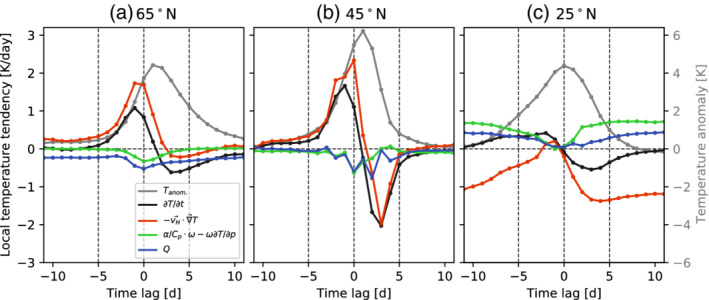
Daily evolution of the temperature tendency equation terms (Equation (7)) for all heatwaves identified occurring at (a) 65°N, (b) 45°N, and (c) 25°N. Each term is averaged between 800 and 1,000 hPa and in a 10° latitude and 20° longitude box centered on the grid points where heatwaves are identified. Using a 10° latitude and 10° longitude box leads to comparable results. The gray line shows the evolution of the temperature anomaly averaged over the same coordinates (y‐axis shown at the right) [Colour figure can be viewed at wileyonlinelibrary.com]

The temporal evolution of the different temperature tendency terms averaged for a box centered on the heatwave location is investigated further in Figure 9. The total temperature tendency starts increasing five days prior to the onset of high‐latitude heatwaves, while this positive temperature tendency starts 10 days prior to subtropical heatwaves (Figure 9a,c). For midlatitude heatwaves, the temperature increases slowly for lags between −10 and −5 and then sharply for the last five days. The first period might be related to a slow increase in the likelihood of atmospheric blocking (shown in Figure 4d), while the latter period tends to be related to the passage of the eastward‐propagating RWP. Note that RWPs and blocking are not independent processes.

Locally, horizontal temperature advection is the main process contributing to the temperature tendency, with the adiabatic and diabatic heating contributing negatively for heatwaves at both 65 and 45°N (Figure 9a,b). This makes sense from a dynamical point of view, as warm air near the surface tends to rise and thus expand adiabatically and cool, which is also shown by Tamarin‐Brodsky *et al*. (2019) when applying a similar analysis for warm temperature anomalies. Therefore, subsidence mostly occurs upstream and downstream of the heatwave region (Figure 7g,k,) and the warmed air is then advected eastward and poleward. An interesting aspect in the evolution of subtropical heatwaves (25°N, Figure 9c) is that they tend to have a slower development than the events occurring further poleward (Figure 9a,b). For subtropical heatwaves, the positive temperature tendency arises from an imbalance between subsidence and advection. Advection, which is climatologically negative at subtropical latitudes (see Figure S2), weakens progressively ahead of the heatwave, and therefore it is no longer able to balance the adiabatic and diabatic heating, thus contributing to a localized warming (Figure 9c). The advection returns quickly to climatological levels 3–5 days after the onset of the heatwave. In contrast, temperature anomalies decay much faster for midlatitude heatwaves (Figure 9b), which are associated with the eastward propagation of a RWP that reverses the sign of the advection term on average two days after the onset of the heatwave. High‐latitude heatwaves, on the other hand, are characterized by a slow decay of the temperature anomaly, as temperature advection does not become sufficiently negative after heatwave onset, and a longer period is needed for diabatic cooling to reduce the temperature anomaly (Figure 9a).

## 6 THE ROLE OF LARGE‐SCALE TOPOGRAPHY

### 6.1 Stationary waves and jet response to idealized topography

Until now, we have used the Held–Suarez no‐topography simulation to isolate the latitude effect on the dynamics and characteristics of heatwaves. However, in the real atmosphere, zonal asymmetries play an important role in setting the preferred position of storm tracks (Kaspi and Schneider, 2013), blocking (Narinesingh *et al*., 2020), and therefore also heatwaves. Zonal asymmetries are introduced in our model through idealized topography of different height at different latitudes (see the methods section 2.2 for details). The main objective is to analyse and characterize the effect of topography on the global distribution of heatwave frequency.

The atmospheric response to idealized topography is examined in terms of the climatological stationary‐wave response: ${\bar{Z}}^{*}=\bar{Z}-[\bar{Z}]$, where Z is the geopotential height at 300 hPa, the overbar represents the climatological mean, and [] is the zonal mean. To characterize the response further, we analyse changes in the upper‐level winds (300 hPa) and blocking frequency by computing the difference between the simulations with topography and the no‐topography simulation. Note that, although our experiments focus on the Northern Hemisphere, the results are equally valid for the Southern Hemisphere.

**FIGURE 10:**
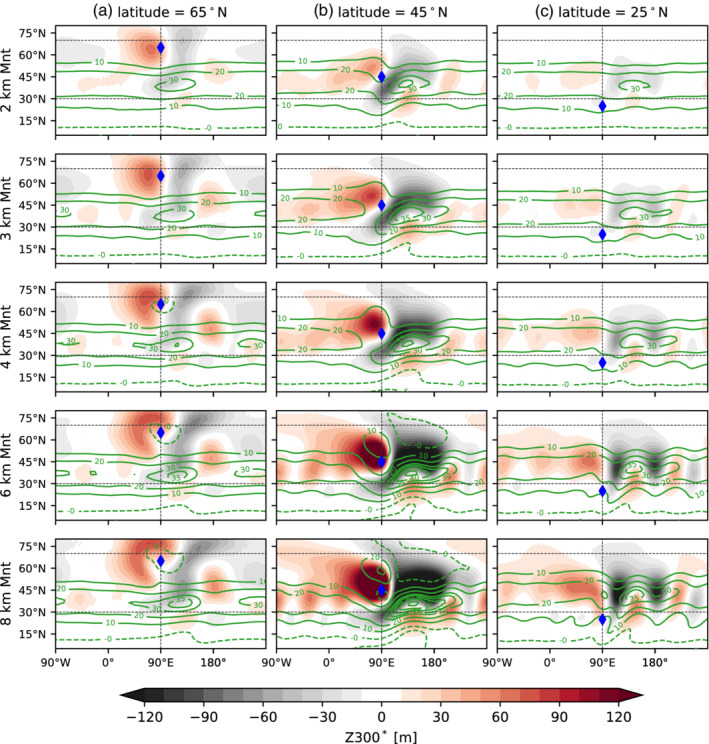
Latitude–longitude distribution of zonal anomaly of geopotential height at 300 hPa (Z300∗, shading) and zonal wind at 300 hPa (U300, green contours) for Held–Suarez model simulations with a Gaussian mountain centered at (a) 65°N, (b) 45°N, and (c) 25°N, and for different mountain heights (increasing mountain height from top to bottom). Anomalies are calculated with respect to the no‐topography simulation. The blue diamond represents the location of the peak of the idealized mountain [Colour figure can be viewed at wileyonlinelibrary.com]

Figure 10 displays the climatological stationary waves ($Z300^{*}$) with respect to the height (H=2,3,4,6, and 8 km) and the latitudinal location of the peak ($\phi_{0}$ = 25, 45, 65°N) of the idealized topography (see Section 2.2). Using different pressure levels in the mid to upper troposphere [500–200 hPa] to define stationary waves does not lead to qualitatively different results. Note that stationary waves in observations or in more complex model simulations result from the nonlinear interaction of topography and other forcings (Garfinkel *et al*., 2020; Wills *et al*., 2019), including latent heat release and surface forcing from land–ocean thermal contrasts. Nonetheless, our purpose here is not to reproduce the exact observed stationary‐wave pattern, but to study the effect on heatwave occurrence when zonal asymmetries in the model climatology are introduced.

The response to a midlatitude mountain located at 45°N is characterized by the formation of a climatological ridge upstream and a climatological trough downstream, leading to a Rossby‐wave train that bends south into the Tropics (Figure 10b). This response is in agreement with results from more complex model experiments using realistic and idealized topography (White *et al*., 2017) and idealized aquaplanet and dry‐core model simulations (e.g., Cook and Held, 1992; Lutsko and Held, 2016; Narinesingh *et al*., 2020). The stationary wave is absorbed in the Tropics, due to the presence of the critical wind line (U=0 line in Figure 10). The upstream anticyclone and downstream cyclone result from PV conservation and vertical deflection of the air columns (Hoskins and Karoly, 1981), that is, the air is forced to ascend on the windward side of the mountain and descends on the leeward side. Horizontal deflection can also occur, which leads to (anti)cyclonic anomalies (north) south of the mountain (Valdes and Hoskins, 1991). This second type of response is more evident for the highest mountains located along the jet‐stream axis (Figure 10b), and is less important for the mountains located north and south of the climatological jet (Figure 10a,c: Lutsko and Held, 2016. Another characteristic is the zonal extent of the cyclonic climatological low downstream of the mountain, which is likely due to enhanced transient eddy feedbacks (Chang, 2009). Our results agree with the nonlinear regime expected for higher mountains (heights above 1 km) found in Lutsko and Held (2016) and Lutsko (2020), who find a similar nonlinear response using a dry general circulation model.

The upper‐level jet accelerates downstream of the mountain (Figure 10b). This acceleration of the zonal winds downstream of the midlatitude mountain is consistent with PV conservation and the thermal wind balance. The surface flow is forced to move around the mountain, which leads to an increased meridional temperature gradient downstream of the mountain and thus an increase with height of the zonal wind (Figure 10b) and increased temperature variability (Lutsko *et al*., 2019).

The stationary‐wave pattern has a strong sensitivity to the latitudinal location of the topographic forcing (different columns in Figure 10). For example, for a mountain located at 65°N (Figure 10a), the stationary‐wave response does not increase considerably in magnitude when increasing the mountain height, whereas for a mountain located at 45°N (Figure 10b) the amplitude of the stationary‐wave pattern increases monotonically with increasing mountain height. A mountain located further south (25°N, Figure 10c) tends to have a much weaker impact on the upper‐level jet and the stationary‐wave pattern compared with a mountain of the same height located further north.

Overall, the strongest stationary wave and jet response takes place when the mountain peak is located along the climatological jet axis (approx. 45°N, Figure 2a), which leads to the strongest impact on the upper‐level jet. This effect does not saturate for the range of topography heights tested here.

### 6.2 Atmospheric blocking and heatwave frequency response to topography

**FIGURE 11:**
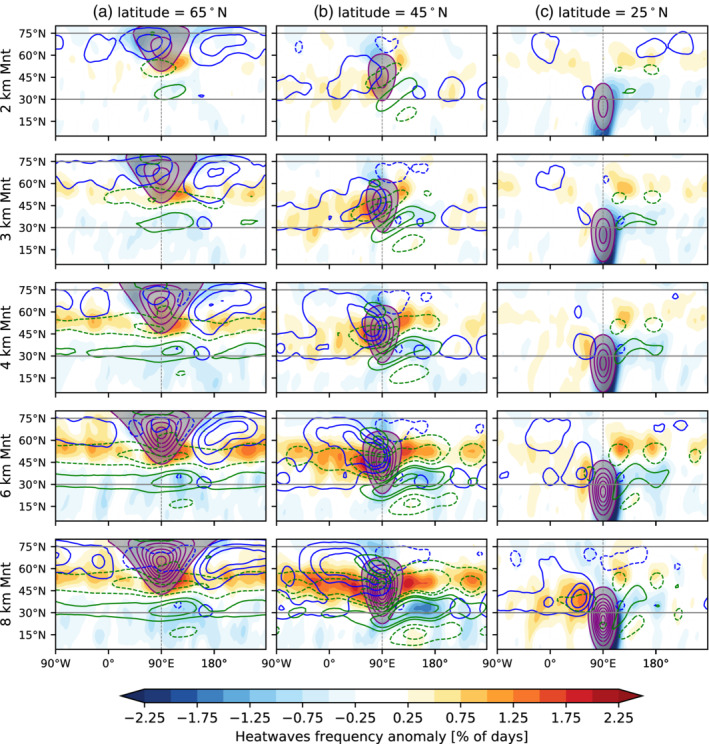
Same as Figure 10 but for the heatwave frequency response (color shading), the 300 hPa zonal wind response (U300, green contours corresponding to −15,−10,−5,5,10,15 m·s${}^{-1}$, where dashed contours correspond to negative values), and blocking anomalies (blue contours corresponding to −2.5,−1.5,−0.5 (dashed) and 0.5, 1.5, 2.5% of blocking days). The topographic elevation is shown in purple contours: from 0.5 to 7.5 km at 1 km intervals, and gray shading masks the elevations above 0.5 km [Colour figure can be viewed at wileyonlinelibrary.com]

We now investigate how zonal asymmetries in the background state impact the frequency of atmospheric blocking and heatwave frequency (Figure 11). Note that we recalculate the 95th percentile used to define heatwaves for each topographic simulation. This is done in order to retain only the contribution from changes in the variability and higher‐order moments of the temperature distribution to heatwaves. If we keep the same threshold as for the no‐topography run, the response is dominated by changes in the temperature climatology, which have been studied extensively (e.g., Cook and Held, 1992). Analysis of the blocking response to topography is motivated by the results found in Section 4 and supported by previous studies linking blocking and heatwaves.

In the Held–Suarez experiments, blocking mostly occurs poleward of the climatological location of the tropospheric jet (see Figure S1c,d, or Figure 2c), which is consistent with the latitude where important blocking centers occur in reanalysis ( Figure S1a,b, representative of Greenland and North Pacific blocking, where cyclonic wave breaking dominates (Masato *et al*., 2013)). For the midlatitude mountain located at 45°N (Figure 11b), the blocking frequency is increased upstream of the mountain and decreased slightly poleward of the mountain. A secondary increase in blocking is located upstream of the topography, at the exit region of the accelerated jet, at latitudes between 30 and 45°N. This effect is much weaker for the 2‐km mountain, but increases considerably with the height of the topography. The upstream positive blocking frequency anomaly increases with the height of the midlatitude mountain (Figure 11b). These results are overall in agreement with the results from Narinesingh *et al*. (2020) using aquaplanet experiments forced with midlatitude topography and using a slightly different definition of blocking.

The latitudinal location of the mountain also influences the blocking response. For example, mountains located closer to the pole (65°N, Figure 11a) lead to increased downstream blocking around 60–75°. Interestingly, blocking increases near the mountain peak for the lower elevations, but decreases for the highest topographic forcing. In contrast, a mountain located south of the tropospheric jet (25°N, Figure 11c) has a much weaker impact on the blocking frequency distribution, with the exception of the 6‐ and 8‐km mountain simulations, which exhibit a large response of subtropical and midlatitude blocking upstream and poleward of the peak.

Consistent with the change in blocking frequency, heatwaves occur more often upstream of the midlatitude mountain than in the no‐topography simulation (Figure 11b). In contrast, fewer heatwaves occur poleward of the mountain peak than without topography. The heatwave response to topography is also characterized by a meridional dipole anomaly downstream, coinciding with the U300 anomalies corresponding to an equatorward shift and strengthening of the upper‐level jet (Figure 11b). The topographic influence tends to expand longitudinally with increasing mountain height both downstream and upstream of the topography. For the extreme case, that is, the 8‐km mountain, the longitudinal extent of the response becomes circumglobal (Figure 11b bottom panel). This increase/decrease of heatwave frequency is highly correlated with the deceleration/acceleration of the upper‐level winds (green contours in Figure 11). A dynamical explanation can be that a weaker jet allows for slower zonal phase and group velocities, that is, synoptic‐scale Rossby waves (k=4–8) tend to propagate more slowly with respect to the surface and thus lead to a higher likelihood of persistent warm extremes (e.g., Fragkoulidis and Wirth, 2020).

For a high‐latitude (65°N) topographic forcing (Figure 11a), the heatwave response is qualitatively similar to the discussed midlatitude forcing, that is, an increased frequency of heatwaves in the midlatitudes, corresponding to a southward shift of the tropospheric jet near the southern edge of the topography and extending both east and west with increasing mountain height (green contours in Figure 11a). The heatwave frequency response is stronger closer to the southern edge of the mountain and downstream, but it extends to the full latitude circle for the strongest topographic forcings (H=4–8 km). This response also closely resembles the negative U300 anomalies for the midlatitude band. In contrast, the blocking response (blue contours in Figure 11a) exhibits a weaker correspondence with the heatwave response, mainly at high latitudes, where the blocking response is strongest but the heatwave frequency response is weak.

The subtropical mountain (25°N) forcing (Figure 11c) leads to a similar, but proportionally much weaker, impact on the heatwave frequency in the midlatitudes (poleward of 30°N) compared with the midlatitude mountain forcing (Figure 11b). Distinct from the response of the more poleward location, there is a localized decrease in the number of heatwaves over the eastern slope of the mountain from 40°N and extending into the Tropics, where easterlies dominate near the surface (Figure 1c), which may be related to more adiabatic cooling on the mountain slope and an accelerated upper‐level jet. For the highest mountain elevations, there is a strong increase of heatwave frequency upstream in the midlatitudes (∼45°N), which coincides with the largest increase of blocking frequency and U300 deceleration (Figure 11c, bottom panel). Further poleward, around 60°N, there are localized areas where the heatwave frequency increases with respect to the no‐topography simulation and mostly coincides with the deceleration of the upper‐level flow (U300).

To summarize, heatwave frequency is modulated by changes in both the blocking and upper winds (U300). While the link between changes in atmospheric blocking and heatwaves is much weaker near the polar regions, we see a stronger correspondence between blocking and heatwave frequency in mid‐ and subtropical latitudes. On the other hand, changes in the upper‐level wind (U300) tend to be more closely connected to the location of heatwaves, as the wind can alter the propagation of synoptic systems causing heatwaves (Fragkoulidis and Wirth, 2020). Interestingly, the RWP response (Figure S9) shows almost the opposite behavior compared with the heatwave frequency in response to zonal wind changes, as RWPs tend to amplify more strongly in regions where the zonal winds are stronger, that is, at the location of the storm track (see figure 4 in Fragkoulidis and Wirth, 2020), which is associated with a decrease in heatwave frequency.

### 6.3 Topographic effect on heatwaves in reanalysis

**FIGURE 12:**
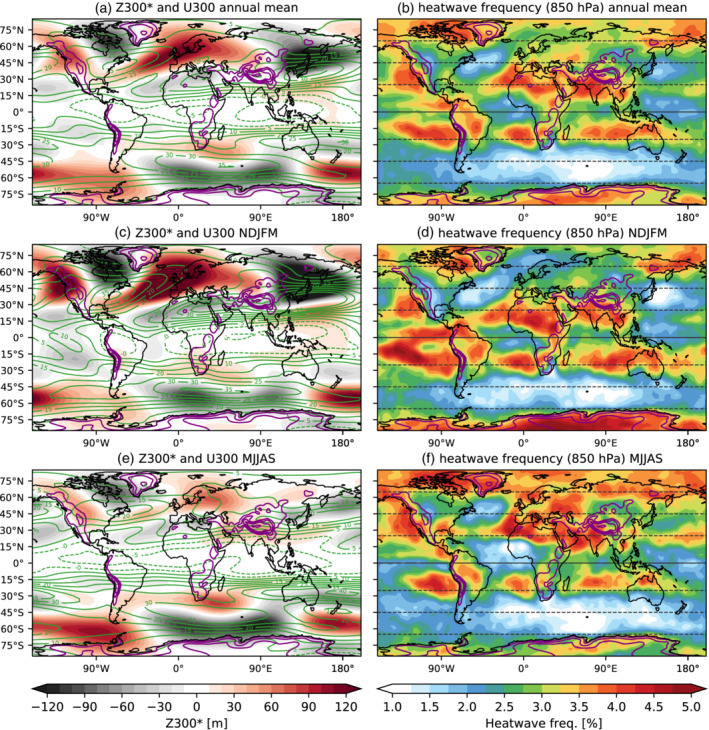
The ERA‐Interim (1979–2019) climatology of (left) $Z300^{*}$ and U300 (as in Figure 10), and (right) heatwave frequency (% of days) computed from the temperature at 850 hPa for (a,b) the annual mean, (c,d) the November–March average, and (e,f) the May–September average. Purple contours show the smoothed elevation (the 1‐, 2‐, 3‐, and 4‐km contours are shown) [Colour figure can be viewed at wileyonlinelibrary.com]

In this section, we compare the idealized model results with the ERA‐Interim reanalysis for 1979–2019. It is important to note that a direct comparison between our model results and the reanalysis is not possible, due to the high degree of idealization. However, some of the conclusions for the pure dynamical response to topography can also be observed in the reanalysis. Figure 12 shows the climatological stationary waves ($Z300^{*}$) and the upper‐level jet (U300), together with the climatological frequency of heatwaves and topographic elevation. We use the same heatwave definition as in the model, but we apply it to the temperature at 850 hPa, as the temperature at this level is more related to the large‐scale circulation and thus this definition represents a more fair comparison with our model results. Nevertheless, using 2‐m temperature instead does not lead to qualitatively different results in terms of heatwave frequency. Boreal winter and summer mean climatologies are shown separately in Figure 12, as climatological waves are characterized by a strong seasonal evolution, especially in the Northern Hemisphere.

One mountain range of particular relevance is the Rocky Mountains (located at $∼[100^{\circ }$W, 45°N] and with a smoothed altitude of around 2,000 m, although local mountain peaks can be much higher). Because this mountain range is located in the midlatitudes, we can see how the jet accelerates (decelerates) downstream (upstream), similar to our 2‐ and 3‐km mountain experiments (Figure 10b), which is linked to an enhanced temperature gradient over North America and enhanced synoptic variability (Lutsko *et al*., 2019). Climatological anticyclonic conditions upstream, that is, over the Northeastern Pacific, which are associated with the changes in the upper‐level jet, favor a higher occurrence of heatwaves, while fewer heatwaves tend to occur downstream of this mountain range due to the accelerated jet (Figure 12a,b). This behavior is found for both summer (Figure 12e,f) and winter (Figure 12c,d), although in boreal summer the North Atlantic jet is located further north, allowing more heatwaves to develop in the southeastern United States (Figure 12f). An example of high‐latitude topography is Greenland, which consists of a thick ice sheet with a peak altitude of above 3 km. In this case, we also observe a higher frequency of heatwaves towards the west rather than the east of the Greenland ice sheet during winter (Figure 12d). However, we cannot attribute the heatwave frequency around Greenland fully to the Greenland ice sheet, as interaction with the other adjacent mountain ranges might be relevant. None the less, White *et al*. (2019) find in model simulations that Greenland plays a key role in the variability of the North Atlantic jet.

Over Asia, the Tibetan Plateau is the largest topographic feature on the planet, located at around 90°E, 30°N and with mean elevations clearly above 5 km. North of this mountain range, the Mongolian mountains ($∼100^{\circ }$E, 45–55°N), despite being lower (H∼1–2 km), exert a greater impact on the North Pacific circulation due to their more poleward location (White *et al*., 2017). As expected from pure dynamical arguments and supported by our idealized simulations, more heatwaves occur upstream than downstream of these two plateaus. For example, heatwaves occur much more frequently over Kazakhstan and Western Russia than over Japan and Northeastern China, where the subtropical jet is strongest (Figure 12a,b), which is consistent with our idealized model simulations. The topographic influence is larger in winter than in summer, when the stationary‐wave response is much weaker (cf. Figure 12c,e). During summer, land–atmosphere feedbacks (Miralles *et al*., 2019) can also contribute strongly to the overall increase of heatwaves over Eurasia (Figure 12f) and therefore this response cannot be attributed solely to the topography.

In the Southern Hemisphere, the Andes ($∼70^{\circ }$W, 0–50°S, mean smoothed elevation ∼3 km) and the elevated Southern African plateau ($∼20^{\circ }$E, 0–30°S, mean smoothed elevation ∼1 km) are the largest elevations. The influence of these mountains ranges, along with the Antarctic ice sheet (Lachlan‐Cope *et al*., 2001) and the effect of tropical sea‐surface temperature (SST) asymmetries (Inatsu and Hoskins, 2004), on the stationary‐wave pattern (Figure 12a) consists of anticyclonic anomalies over the South Pacific sector and cyclonic anomalies downstream of the Andes in the South Atlantic and Indian Ocean sectors. The upper‐level jet is also stronger downstream of the Andes, which coincides with the area with the lowest frequency of heatwaves (Figure 12a,b). On the other hand, more heatwaves occur upstream of these two mountain ranges. The Andes can also influence the Southern Hemisphere stationary‐wave pattern through its indirect influence on the tropical Pacific circulation (Takahashi and Battisti, 2007).

The role of latitude that we have identified in our idealized simulations can also be observed in the reanalysis, where subtropical and high‐latitude regions tend to show a higher frequency of heatwaves, in contrast to the midlatitudes (compare Figure S4d and 2d). Latitude and topography are just two main factors explaining the distribution of heatwaves on the planet, and other factors like land and ocean interactions exert very important roles (e.g., Behera *et al*., 2013; Duchez *et al*., 2016; Ossó *et al*., 2020; Seo *et al*., 2020). Our idealized simulations and analysis show that heat extremes can be attributed to different underlying dry dynamics, depending on the latitude where they occur, and that topography plays an important role in setting the mean distribution of persistent heat extremes.

## 7 SUMMARY AND DISCUSSION

The main objectives of this study are two‐fold: first, to understand and isolate the role of dry atmospheric dynamics in heatwave evolution and characteristics at different latitudes, and, second, to study and characterize the role of large‐scale topography in heatwave frequency and distribution when only dry atmospheric dynamics are considered. To understand the role of atmospheric dynamics, we use idealized atmospheric model experiments where physical parameterisations are substituted by a simple temperature relaxation scheme, as in Held and Suarez (1994). In particular, to study the effect of latitude on heatwaves we use a zonally symmetric model simulation without topography. To gain a deeper insight into the physical processes leading to the extreme warming associated with heatwaves, we calculate the terms of the temperature tendency equation. Finally, we quantify the heatwave frequency and atmospheric circulation changes in response to idealized topography of different height and located at different latitudes. Note that, even though we have analysed results for the Northern Hemisphere, our results are equally valid for the Southern Hemisphere, given the idealized nature of our model experiments. The results of our analysis can be summarized as follows.

1. The dynamical evolution and main characteristics (duration, intensity, and horizontal extent) of heatwaves depend on the latitude where these occur (Figure 5). While midlatitude heatwaves are associated with the eastward propagation and local amplification of Rossby‐wave packets, together with an enhanced probability of blocking (Figure 4d), atmospheric blocking is identified as the dominant circulation driver for heatwaves occurring in high latitudes (Figure 4b). In contrast, blocking is, in general, not involved in the evolution of subtropical heatwaves (Figure 4f). For subtropical heatwaves, a strong meandering of the midlatitude jet associated with a localized anomalous surface low‐pressure system east of the heatwave location is the large‐scale circulation pattern linked to heatwave development (Figure 3, bottom row).
2. Horizontal temperature advection is the process that accounts for most of the temperature increase for high‐ and midlatitude heatwaves occurring in the dry dynamical core simulations, while adiabatic and diabatic processes have a small negative contribution (i.e., a cooling effect: Figure 9a,b). In contrast, adiabatic heating due to large‐scale subsidence dominates the slower temperature increase associated with subtropical heatwaves (Figure 9c). For subtropical heatwaves, although the total horizontal advection has a cooling effect, a linear positive anomaly of this term (less cooling) leads to a local imbalance with adiabatic and diabatic heating, and thus to warming (Figure 9c). The role of advection in driving heat extremes is consistent with the results from Linz *et al*. (2018), who find in a very simple model that the skewness of the temperature distribution is largely controlled by eddy stirring.
3. The mean heatwave duration exhibits a minimum in midlatitudes and increases poleward and equatorward of the climatological jet stream (Figure 5a). In contrast, the mean and maximum intensity of heatwaves exhibits the opposite behavior, that is, midlatitude heatwaves tend to be more intense than heatwaves occurring further poleward and equatorward (Figure 5b,c). For all latitudes considered here, the mean intensity and duration of dynamically driven heatwaves are independent of each other, that is, a more intense heatwave is not necessarily longer (Figure 6).
4. In general, mountains located along the midlatitude jet axis have a stronger impact on the stationary‐wave pattern and the upper‐level jet response than mountains equatorward and poleward of the jet axis (Figure 10). Upstream, a deceleration of zonal winds and increased blocking frequency upstream of the topography are likely driving the increase in heatwaves, while an accelerated upper‐level jet leads to less persistent extremes and thus a decrease in heatwaves equatorward and downstream of the midlatitude topography (Figure 11b). For the highest mountain elevations located in mid and high latitudes, the heatwave response is circumglobal, characterized by a general increase of heatwaves in the latitude band 45–65°N (Figure 11a,b). In contrast, the effect of topography located south of the jet stream is much weaker, with the largest heatwave frequency corresponding to enhanced blocking probability.
5. The climatology of heatwave frequency in the ERA‐Interim reanalysis shows a similar dependence on latitude to our model simulations (Figure 12). The effect of Earth's main mountain ranges can also be identified: for example, as larger heatwave frequency upstream compared with downstream of the Rocky Mountains, the Mongolian and Tibetan plateaus, or the Greenland ice sheet.

While this study focuses on isolating the dry dynamical contribution to heatwave evolution and properties, in the real world water vapor can play an important role. Latent heat release in warm conveyor belts has been found to be an important factor for atmospheric blocking (Pfahl *et al*., 2015; Steinfeld *et al*., 2020). It is expected that if blocking becomes more persistent then polar and midlatitude heatwaves would also become more persistent. However, it is less clear how atmospheric water vapor can impact the intensity of heatwaves. Recent studies focusing on Greenland ice‐melting events (Hermann *et al*., 2020) and Arctic heatwaves (Murto *et al*., 2021) identify latent heating of air parcels as a large contributor to polar heat extremes. Therefore it is expected that, if water vapor were included in our model simulations, polar heatwaves would become more intense.

Our temperature tendency results are also in stark contrast with the findings of Zschenderlein *et al*. (2019), who conclude that horizontal temperature advection is “negligible” and “not an important” driver for summer European heatwaves. This discrepancy can be explained through the fundamental differences between the study of Zschenderlein *et al*. (2019) and the present one. First of all, Zschenderlein *et al*. (2019) focus on summer European heatwaves, while here we analyse idealized model simulations, which do not represent continents and do not realistically represent diabatic effects like shortwave radiation, which in summer can have a strong effect in warming the surface over continental regions like Europe and can lead to large surface sensible heat fluxes. The other important difference between the two studies is the Lagrangian approach used in Zschenderlein *et al*. (2019), which accounts for diabatic and adiabatic warming along the trajectory of air parcels, while we use an Eulerian approach. Therefore, while our results might not apply to summer heatwaves over Europe in particular, they provide us with a better understanding of the latitudinal dependence of large‐scale systems leading to heat extremes in the absence of diabatic processes.

Furthermore, the effects of ocean and land‐surface properties have been excluded intentionally in our model experiments. Our approach allows us to isolate the pure effect of atmospheric dynamics on heatwaves. However, in the real world, land–atmosphere feedbacks play an important role and have been suggested to enhance the persistence and intensity of heatwaves (e.g., Miralles *et al*., 2019). SST anomalies have also been linked to land heatwaves (Duchez *et al*., 2016; McKinnon *et al*., 2016; Mecking *et al*., 2019; Finke *et al*., 2020). Tropical SST anomalies can force tropical–extratropical teleconnections, which, together with nonlinear transient eddy feedbacks, also play an important role in maintaining stationary‐wave anomalies leading to extratropical heatwaves (Wulff and Domeisen, 2019; Ma and Franzke, 2021).

The role of the seasonal cycle has been excluded in our model simulations. As discussed in the Introduction, atmospheric blocking can lead to opposite effects over land in winter and summer (Nabizadeh *et al*., 2021; Sousa *et al*., 2018). In observations, the main mechanism behind the seasonal difference in the response to blocking is through changes in the effect of radiation on surface and diabatic processes. The Held–Suarez setup used here does not represent the radiative transfer and its impact on temperature extremes explicitly; therefore our model configuration is useful to assess the direct links between circulation and heat extremes excluding the diabatic contribution. Moreover, we analyse the characteristics of heatwaves occurring poleward and equatorward of the midlatitude jet, the location of which in the real world depends on the seasonal cycle and determines the large‐scale dynamics that shape the evolution of heat extremes.

In conclusion, this study describes and characterizes the role of latitude and topographic forcing in shaping the characteristics of heat extremes. Future work might focus on the climate‐change impact on the dynamics associated with heatwaves in idealized model experiments. Previous work using model configurations similar to the one employed here finds a decrease in blocking occurrence when the meridional temperature gradient decreases as a result of Arctic amplification (Hassanzadeh *et al*., 2014). On the other hand, several studies suggest that heatwave frequency, intensity, and persistence will increase in a warmer climate (Meehl and Tebaldi, 2004; Perkins‐Kirkpatrick and Gibson, 2017; Perkins‐Kirkpatrick and Lewis, 2020; Suarez‐Gutierrez *et al*., 2020; Kornhuber and Tamarin‐Brodsky, 2021) and that soil‐moisture‐driven heat extremes could become more common in a future warmer climate (Vogel *et al*., 2017; Suarez‐Gutierrez *et al*., 2020). This indicates that the role of atmospheric dynamics in the future climate is still debated (Shepherd, 2014), and therefore a detailed understanding of the relevant dynamical processes driving heatwaves is crucial for an understanding of future extremes.

## AUTHOR CONTRIBUTIONS

**Bernat Jiménez‐Esteve:** conceptualization; data curation; formal analysis; investigation; methodology; software; visualization; writing – original draft; writing – review and editing. **Daniela I.V. Domeisen:** conceptualization; funding acquisition; supervision; writing – review and editing.

## CONFLICT OF INTEREST

The authors declare that they have no conflict of interest.

## Supporting information


**Appendix S1**. Supporting information.
