# Supplementary material for: The role of atmospheric dynamics and large‐scale topography in driving heatwaves
Source: Q J R Meteorol Soc. 2022 Jul 7;148(746):2344–67. doi: 10.1002/qj.4306 (PMC9544589; doi:10.1002/qj.4306)
Supplement: Supplementary file 1 — Appendix S1. Supporting information. [file QJ-148-2344-s001.pdf]

Supplementary material for the paper "The role of atmospheric dynamics and large-scale topography in driving heatwaves"

B. Jiménez-Esteve and D. I. V. Domeisen

1 Introduction

This supplementary material contains extra information, including text and figures to support the analysis of the main manuscript entitled "The role of atmospheric dynamics and large-scale topography in driving heatwaves". This includes an in-depth analysis of the temperature tendency equation and its linearised form.

Supplementary figures are also displayed at the end of this document in the same order that they are referred to in the main text.

2 Supplementary text

2.1 Temperature tendency zonal mean climatology

Figure S2 displays the zonal mean climatology of the horizontal advection ($-u\frac{\partial T}{\partial x} - v\frac{\partial T}{\partial y}$), the adiabatic ($-\omega\frac{\partial T}{\partial p} + \frac{1}{\rho C_p}\omega$) and diabatic ($Q$) terms of the temperature tendency equation (eq. 7 in the main text). Note that we combine the vertical advection term with the adiabatic warming, as they tend to compensate each other. Furthermore we compute these terms for each grid point (x,y,p) before zonally averaging our results, in order to retain the contribution from eddies. In general, horizontal advection transports heat from the tropics to the extratropics (Figure S2a). Note that the zonal component of advection averages out when zonally averaged. Adiabatic heating mostly occurs in the subtropics and maximizes near the surface around 900 hPa, consistent with the climatological subtropical subsidence in the model, while rising air linked to the Hadley cell and in the storm track region leads to climatological adiabatic cooling at the equator and in midlatitudes, respectively (Figure S2b). Diabatic heating results from the Held-Suarez temperature relaxation and leads to strong heating near the surface in the tropics [40°S,40°N] and cooling in the polar regions, thus counteracting horizontal advection (which includes the poleward heat transport by eddies and the meridional mean circulation). Climatologically the three terms represented in Figure S2a-c have to cancel each other as the simulation is in equilibrium ($\partial T/\partial t = 0$ when averaged over all the simulation days).

We also analyze how much each of these terms contributes to local heating of the atmosphere

under moderate heating (see definition below), which is a necessary condition for the genesis of a heatwave (Figure 9). For each grid point (x,y,p) separately, we compute the composite mean for days where the total temperature tendency is above 1K/day (Figure S2d-f) and then zonally average the results. Horizontal advection is the main contributor to warm tendencies in the extratropics, with a maximum located in midlatitudes (∼45°) and near the surface. In contrast, in the tropics (<30°), adiabatic warming is the most important process that leads to substantial heating, which coincides with the climatological descending branch of the Hadley cell. Diabatic heating is the least important process in this model configuration, important only very close to the surface and in the tropics, although essential to maintain the meridional temperature gradient.Diabatic heating is not a significant contributor to warm tendencies over most of the domain, but notably it is the dominant driver close to the surface in the topics.

The same analysis as in Figure S2 is repeated for the ERA-Interim reanalysis (Figure S3). Despite some evident differences, mainly in terms of the diabatic heating, our idealized simulations reproduce a very similar structure and magnitude of the horizontal advection and adiabatic heating for the warm tendency days. One clear difference is the importance of diabatic heating within the Inter-tropical convergence Zone (ITCZ) and the storm track region due to latent heat release, which is not simulated in our model. Nevertheless, despite the high level of idealization of our model experiments, the two main physical processes leading to temperature extremes in the main regions of interest for this study, i.e. the subtropical and extratropical regions [65-25°], are well represented.

2.1.1 Linearisation of the Temperature tendency

The linearised temperature tendency equation takes the following form:

$$\frac{\partial T}{\partial t} = -\vec{u}_c\frac{\partial T_c}{\partial x} - \vec{u}_a\frac{\partial T_a}{\partial x} - \vec{u}_c\frac{\partial T_a}{\partial x} - \vec{u}_a\frac{\partial T_c}{\partial x} - \omega_c\frac{\partial T_c}{\partial p} - \omega_c\frac{\partial T_a}{\partial p} - -\omega_a\frac{\partial T_c}{\partial p} - \omega_a\frac{\partial T_a}{\partial p} + \frac{1}{\rho C_p}\omega_a + \frac{1}{\rho C_p}\omega_c + Q \quad (1)$$

where the sub-index a indicates the anomaly components and the sub-index c the climatology of each term. Note that we have used the $\vec{u} = (u, v)$ convention to simplify the terms.

All terms containing only interaction between climatological components are climatological terms, and represent the effect of the mean climatological circulation:

$$\left.\frac{\partial T}{\partial t}\right|_{\text{clim}} = \underbrace{-\vec{u}_c\frac{\partial T_c}{\partial x}}_{\text{h. advec.}}\underbrace{-\omega_c\frac{\partial T_c}{\partial p} + \frac{1}{\rho C_p}\omega_c}_{\text{adiabatic}} \quad (2)$$

All terms that contain only one anomaly component are linear terms and represent the eddy-

mean flow interactions:

$$\left.\frac{\partial T}{\partial t}\right|_{\text{lin}} = \underbrace{-\vec{u}_c\frac{\partial T_a}{\partial x} - \vec{u}_a\frac{\partial T_c}{\partial x} - \vec{u}_a\frac{\partial T_a}{\partial x}}_{\text{horiz. advec.}} \underbrace{-\omega_a\frac{\partial T_c}{\partial p} - \omega_c\frac{\partial T_a}{\partial p} + \frac{1}{\rho C_p}\omega_a}_{\text{adiabatic}} \tag{3}$$

Finally, all terms involving the interaction of two anomaly components are called nonlinear terms (eddy-eddy interactions):

$$\left.\frac{\partial T}{\partial t}\right|_{\text{non-lin}} = \underbrace{-\vec{u}_a\frac{\partial T_a}{\partial x}}_{\text{h. advec.}} \underbrace{-\omega_a\frac{\partial T_a}{\partial p}}_{\text{v. advec.}} \tag{4}$$

The linearised equation 4 consists in eliminating all the nonlinear terms. However, due to the large importance of the nonlinear interactions we also show the nonlinear components for the composite of heatwaves in Figures S6, S7 and S8.

We calculate the 30-year climatology of u, v, ω and T for each grid point and pressure level for the no-topo ICON simulation. The daily anomalies u_a, v_a, ω_a and T_a are then calculated by subtracting the climatological values to the daily mean values. Then each term in the temperature tendency equation is computed using centered finite differences. The climatological terms (eq. 2), the linear terms (eq. 3) and nonlinear terms (eq. 4) are composited with respect the onset of the heatwaves as in Figures 7 and 9 in the main manuscript. The diabatic heating is computed as a residual and therefore it is not possible to decompose it in the different contributions.

Figure S6 displays the decomposition of the horizontal advection. We can see that the linear horizontal advection dominates in the midlatitude (Figure S6g) and subtropics (Figure S6k), while nonlinear advection has a larger contribution to the temperature increase for high-latitude heatwaves (Figure S6d). Interestingly the nonlinear advection (eddy-eddy interactions) is negative south of the climatological location of the eddy-driven jet and therefore has a negative contribution to subtropical heatwaves (Figure S6l). The climatological advection has only a slightly positive contribution to the midlatitude heatwaves (Figure S6l), while it is slightly negative for subtropical heatwaves due to the dominant trades winds in the tropics (Figure S6j). The temporal evolution of these terms is displayed in Figure S8.

Figure S7 displays the decomposition of the adiabatic and vertical advection terms. We find that the weak negative adiabatic heating for high- and midlatitude heatwaves is mostly coming from the eddy-mean flow interaction (linear terms, Figure S7g,k). The linear contribution displays a wave train structure, which is consistent with the quasi-geostrophic theory. Important to highlight is the large contribution of the climatological adiabatic warming for subtropical heatwaves, which are located at the location of largest subsidence associated with the Hadley cell (Figure S7j). The climatological adiabatic warming is counteracted with enhanced negative contribution form the linear term (Figure S7k).

To summarize these results, Figure S8 displays the temporal evolution for the different terms

as in Figure 9 of the main text. Interesting to note is the dominant contribution of nonlinear advection for high-latitude heatwaves, which quickly changes sign after the onset of the heatwave and is counteracted by the linear advection, which remains positive for longer (Figure S8a). In contrast nonlinear advection only becomes slightly positive for midlatitude heatwaves at the onset of the midlatitude heatwaves, and linear advection is the dominant process driving the temperature increase (Figure S8b). For subtropical heatwaves, interesting to note is the opposite sign of the linear and nonlinear horizontal. advection terms (Figure S8c).

Supplementary figures:

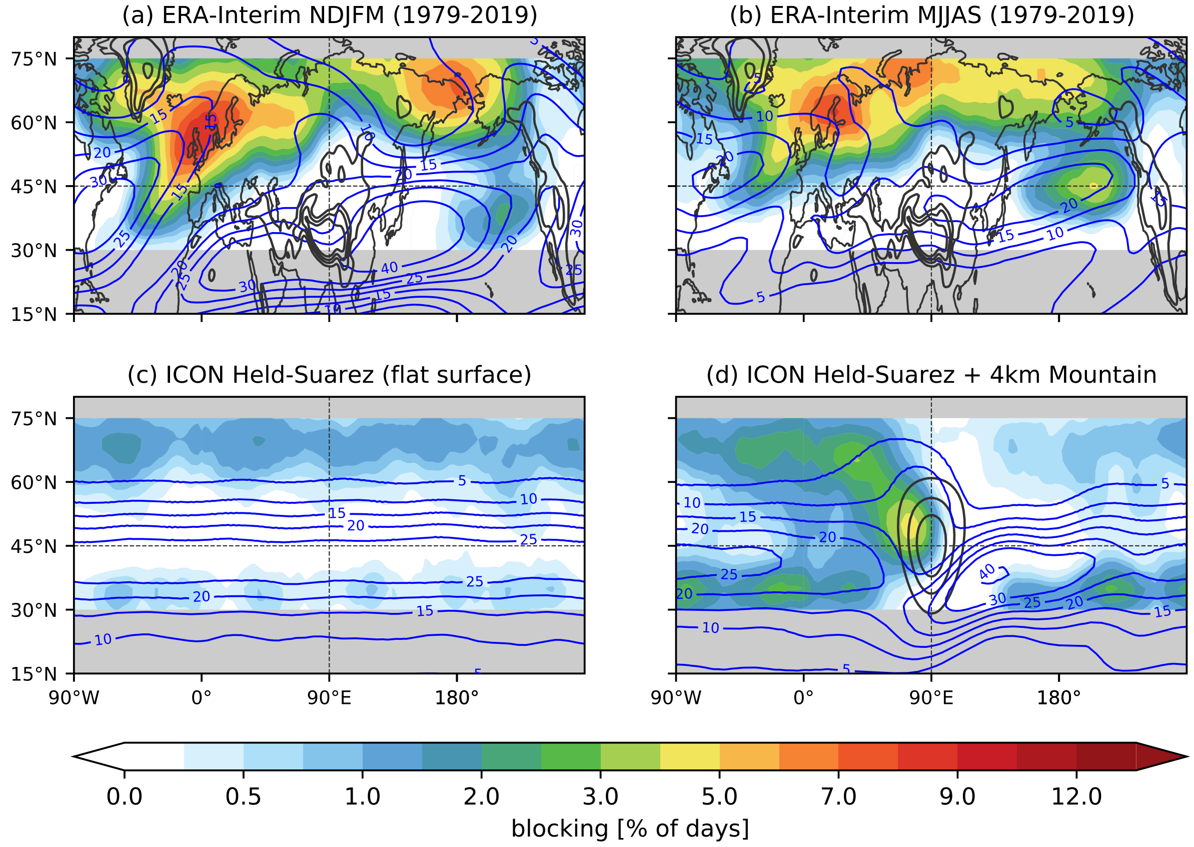

Figure S1: Blocking frequency (shading), calculated following the methods described in this paper, for (a) November to March (NDJFM) and (b) May to September (MJJAS) ERA-Interim reanalysis, (c) the Held-Suarez no-topography simulation, and (d) with a 4 km Gaussian mountain located at (45°N,90°E). Blue contours represent the zonal wind at 300 hPa, and black contours the surface height (1, 2, 3, 4 km are displayed)

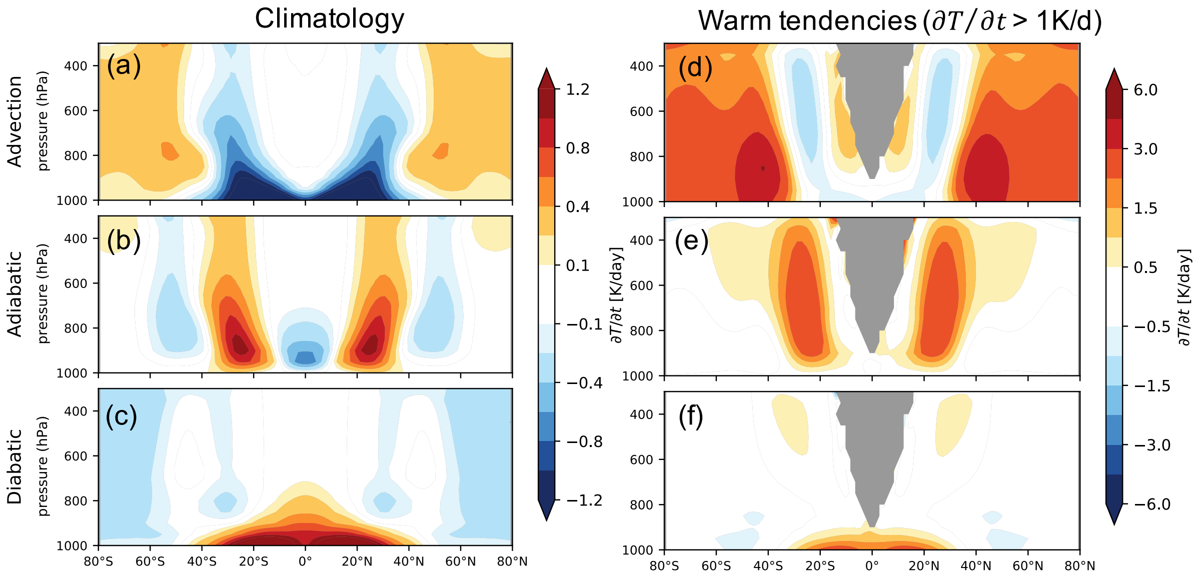

Figure S2: (a-c) Climatology of the temperature tendency terms (equation 6) computed for the Held-Suarez no-topography experiment. (d-f) The same terms but averaged over days with moderate and strong warm tendencies (>1K/day). Grey shading areas in (d,e,f) represent locations where the previous threshold is never reached.

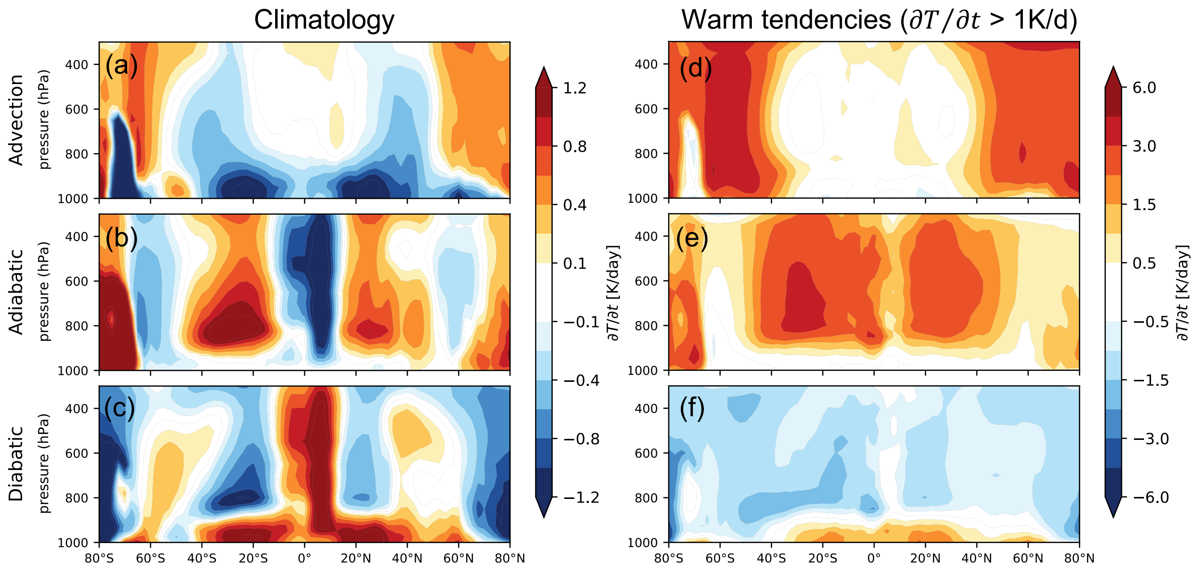

Figure S3: Same as Figure S2 but for the annual ERA-Interim reanalysis (1979-2019).

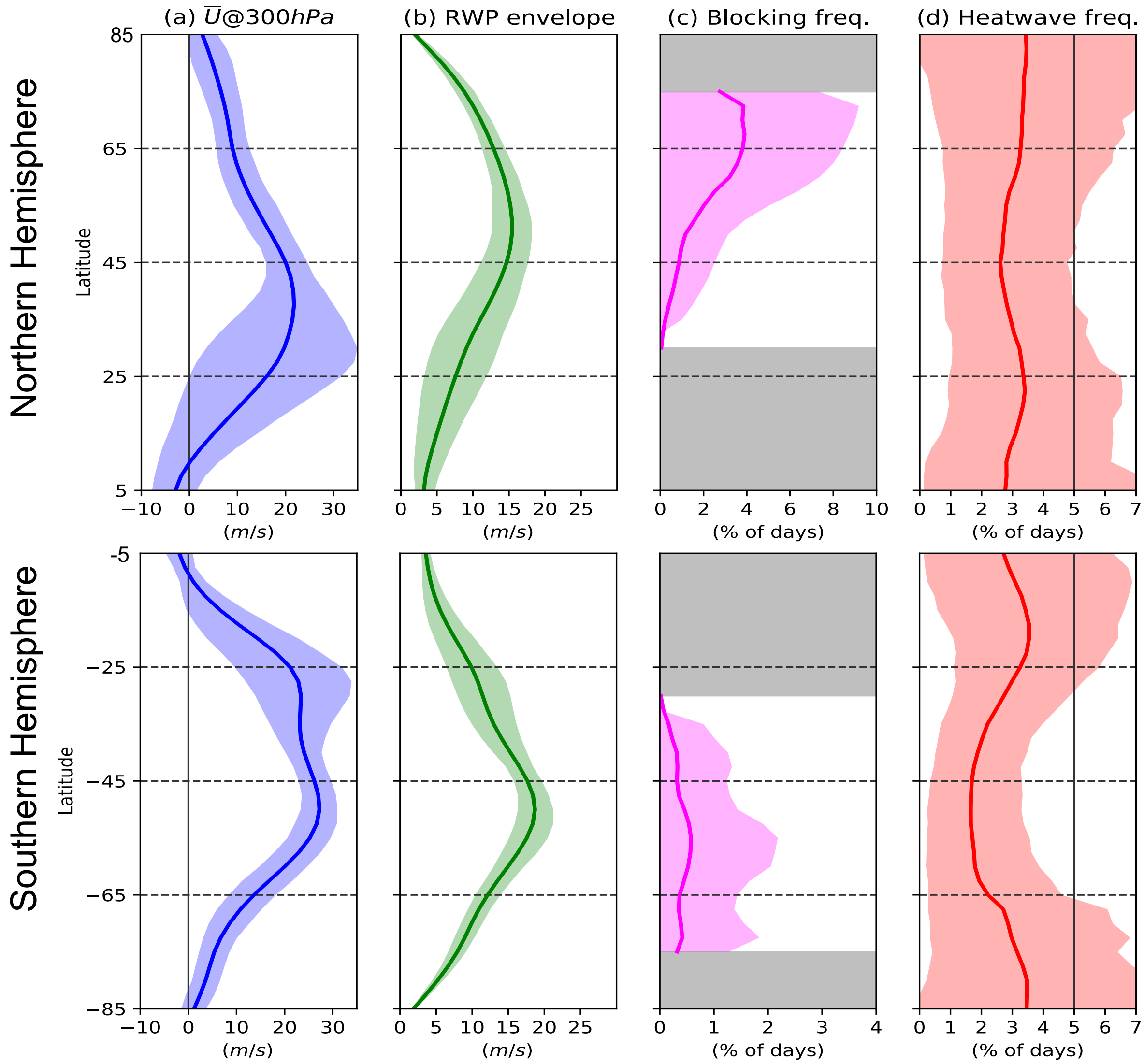

Figure S4: Same as in Figure 2 of the main text, but for the ERA-Interim reanalysis (Jan 1979-Aug 2019). Top row: Northern Hemisphere, Bottom row: Southern Hemisphere. Note the different x-axis for blocking frequency in the Northern and Southern Hemispheres.

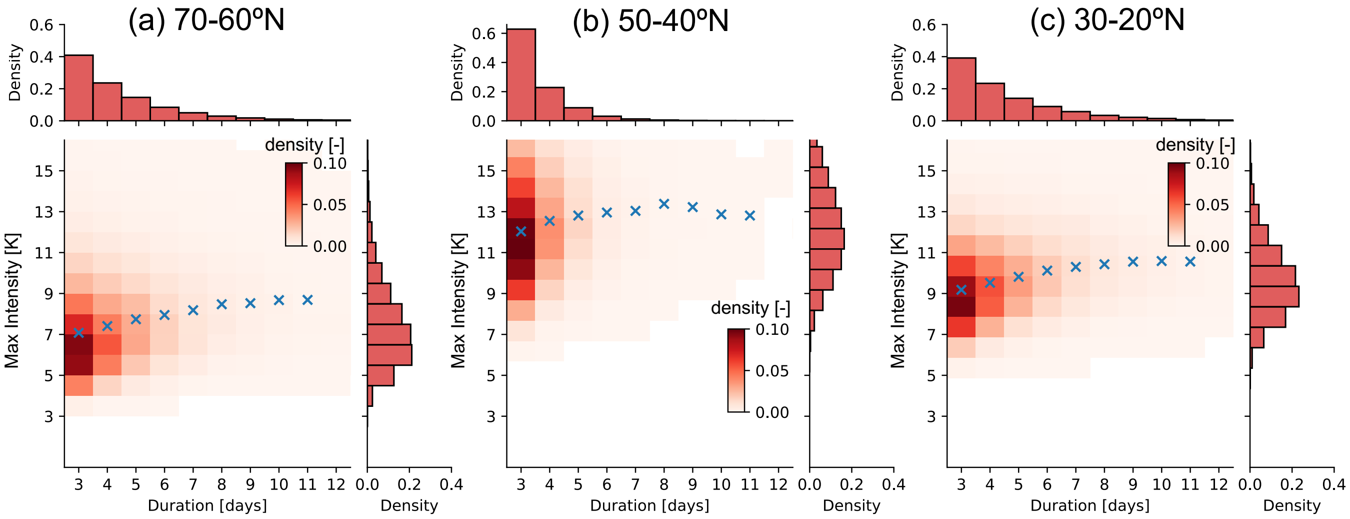

Figure S5: Same as in Figure 6 of the main text but instead of mean intensity, maximum intensity is shown on the y-axis.

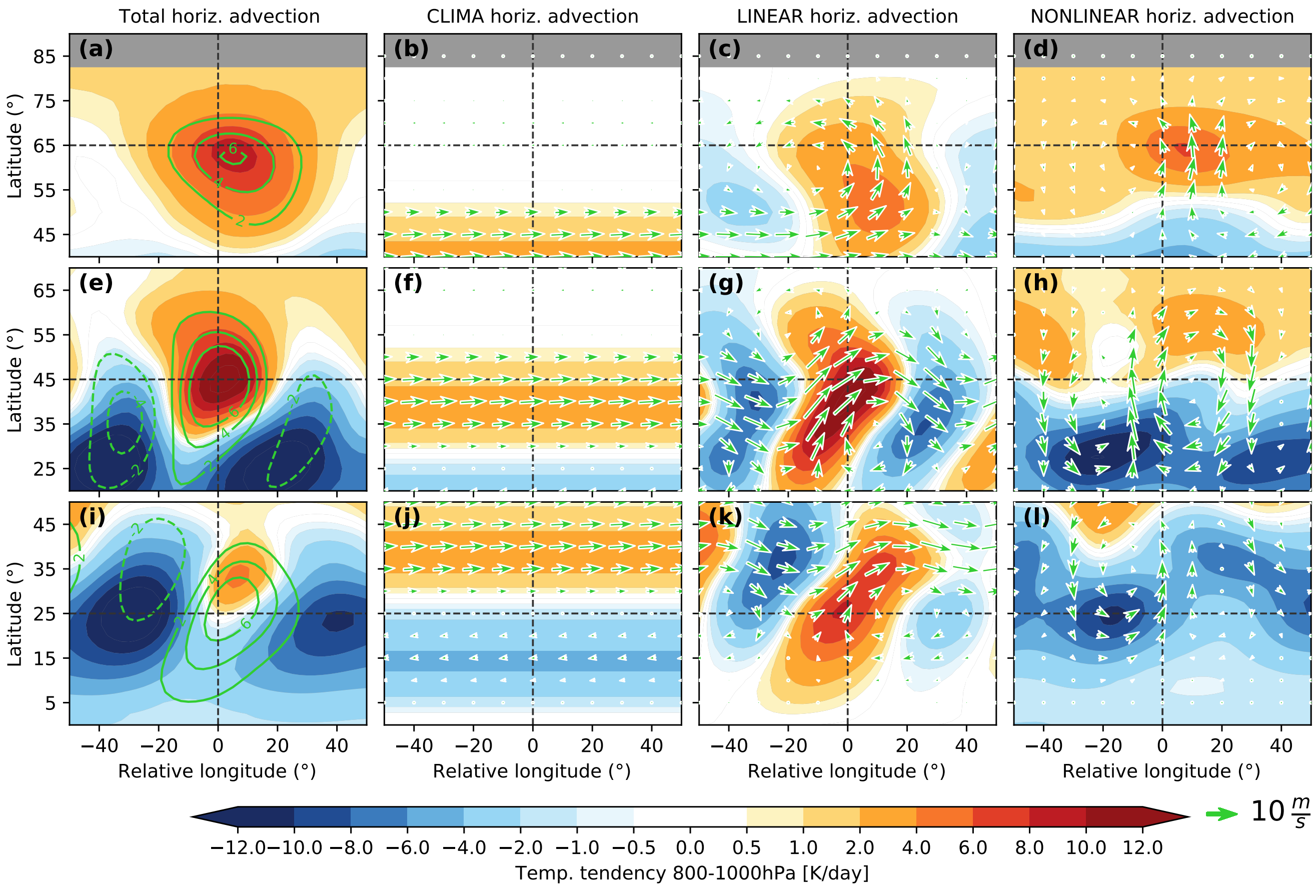

Figure S6: Similar to Figure 7 in the main text, but for the decomposition of the (a,e,i) horizontal advection term into (b,f,j) climatological, (c,g,k) linear, and (d,h,l) nonlinear contributions. The vectors in (b,f,j) indicate climatological, in (c,g,k) the total, and in (d,h,l) the anomalous horizontal wind vectors at 850hPa. The methods can be found in this supplementary material.

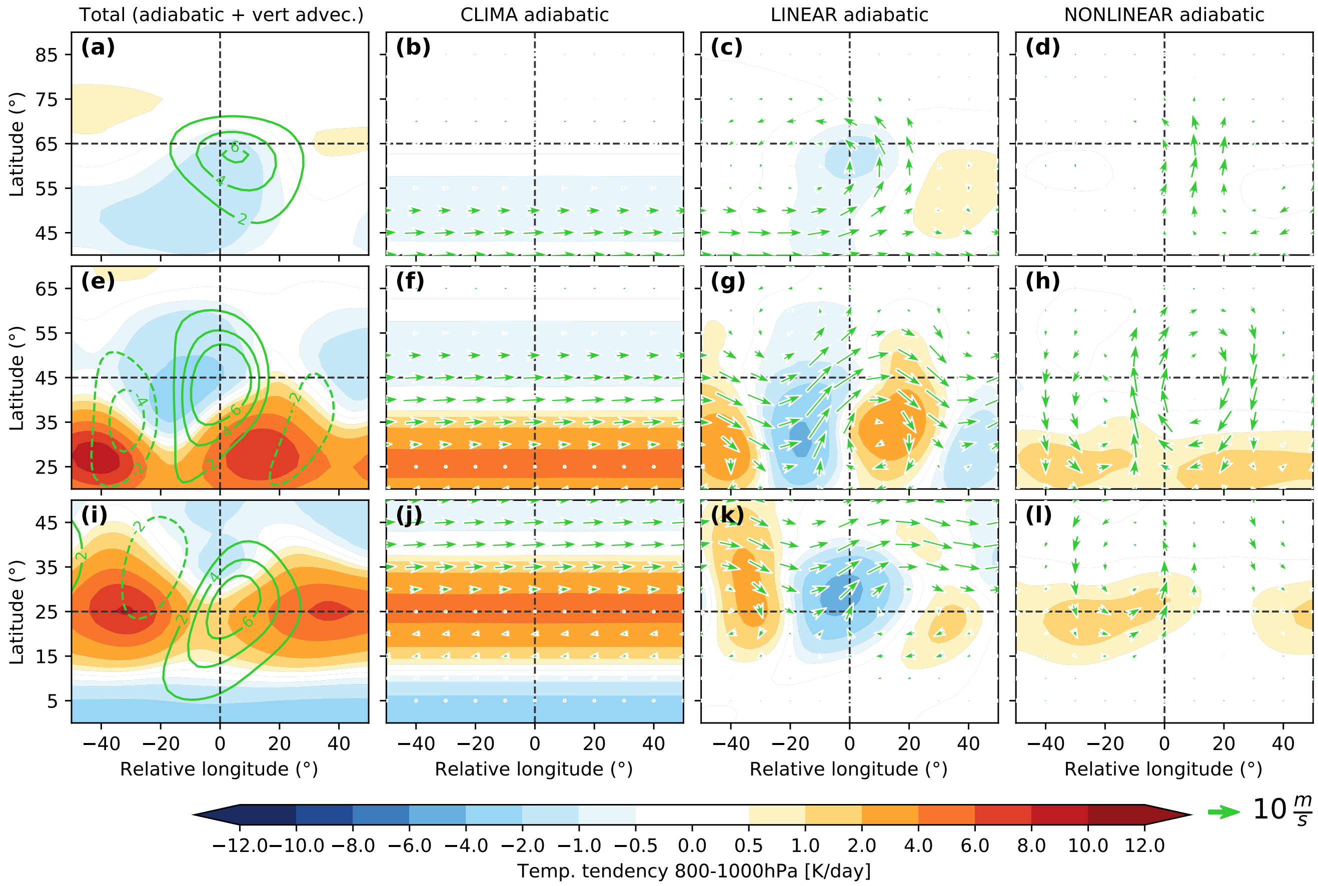

Figure S7: Similar to Figure 7 in the main text, but for the decomposition of the (a,e,i) adiabatic term into (b,f,j) climatological, (c,g,k) linear, and (d,h,l) nonlinear contributions. The vectors in (b,f,j) indicate climatological, in (c,g,k) the total, and in (d,h,l) the anomalous horizontal wind vectors at 850hPa. The methods can be found in this supplementary material.

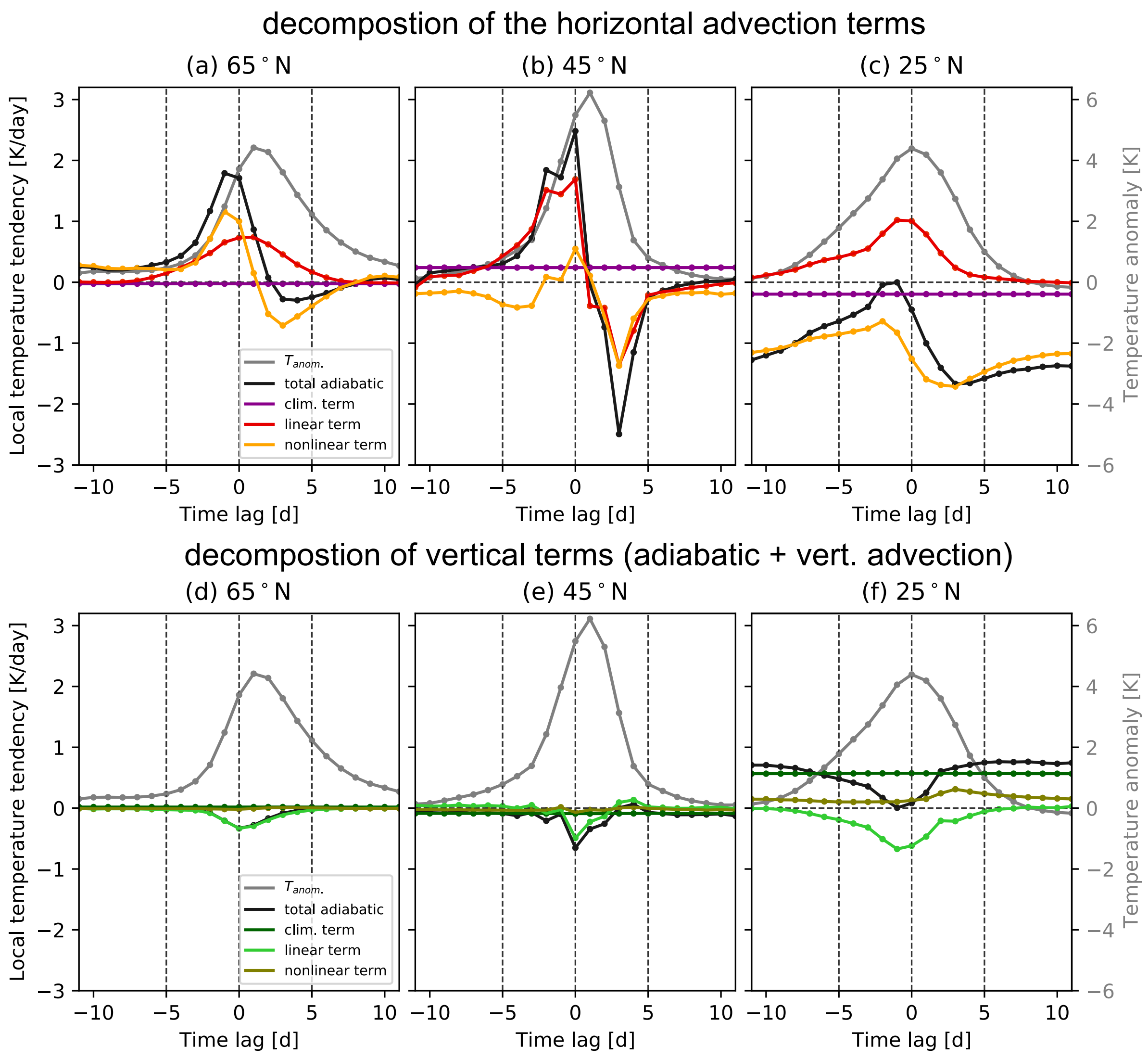

Figure S8: Same as in Figure 9 of the main text but showing the decomposition into the climatological, linear, and nonlinear contributions of the (a-c) horizontal advection term, and (d-f) adiabatic term.

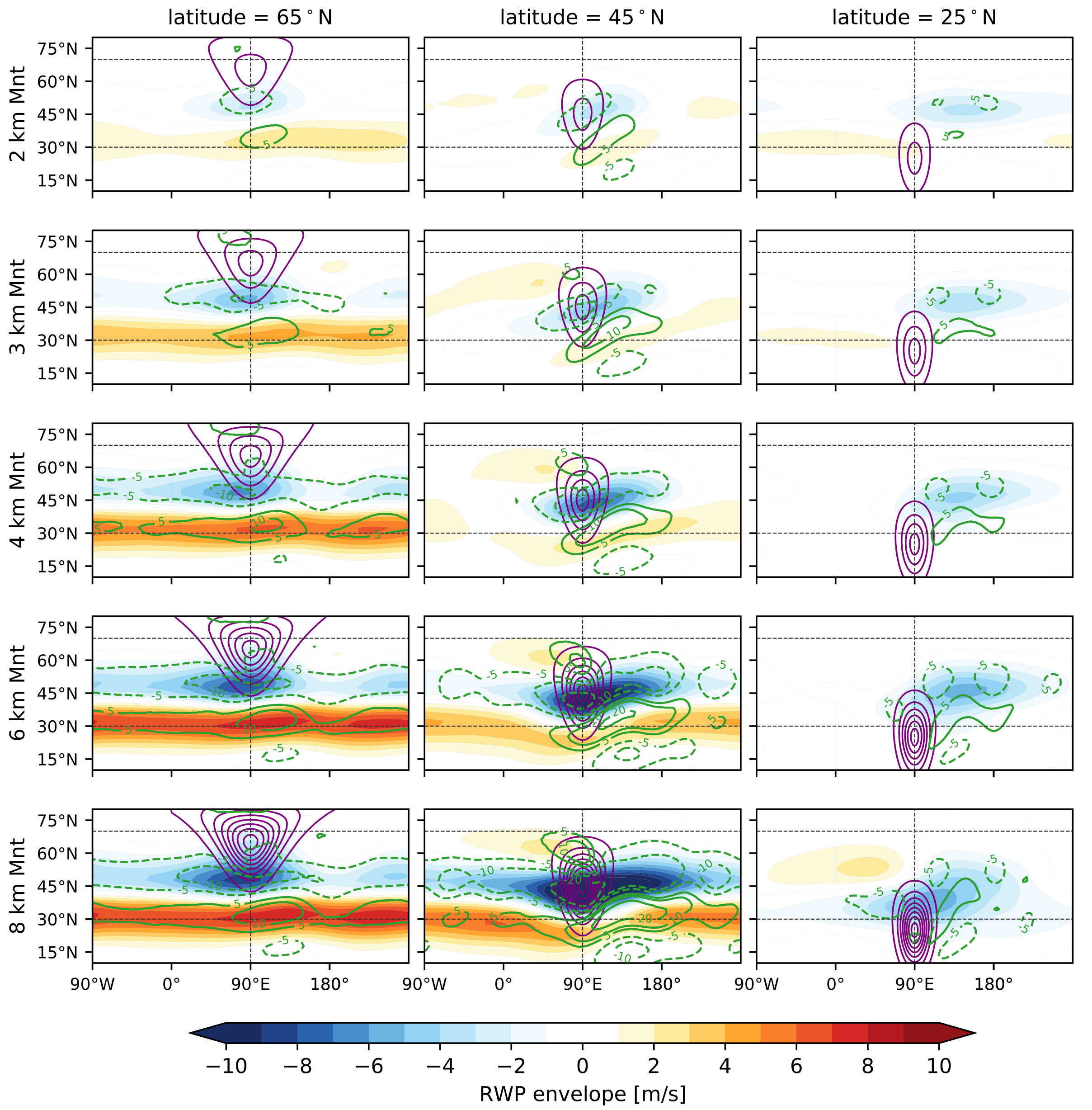

Figure S9: Similar to Figure 10 of the main text but for the RWP envelope at 300 hPa response (color shading) and zonal wind anomalies (U300, shading).
